# A Mutated Nme1Cas9 Is a Functional Alternative RNase to Both LwaCas13a and RfxCas13d in the Yeast *S. cerevisiae*

**DOI:** 10.3389/fbioe.2022.922949

**Published:** 2022-06-02

**Authors:** Yadan Zhang, Huanhuan Ge, Mario Andrea Marchisio

**Affiliations:** School of Pharmaceutical Science and Technology, Tianjin University, Tianjin, China

**Keywords:** CRISPR-Cas, RNase, *S. cerevisiae*, synthetic biology, mRNA degradation

## Abstract

CRISPR–Cas systems provide powerful biological tools for genetic manipulation and gene expression regulation. Class 2 systems, comprising type II, type V, and type VI, have the significant advantage to require a single effector Cas protein (Cas9, Cas12, and Cas13 respectively) to cleave nucleic acids upon binding the crRNA. Both Cas9 and Cas12 recognize DNA and induce a double-strand break in it. In contrast, Cas13 bind and cleave RNA exclusively. However, some Cas9 homologs have shown RNase activity as well. Here, we harnessed Nme1Cas9, LwaCas13a, and RfxCas13d to carry out gene downregulation in *Saccharomyces cerevisiae* by triggering mRNA degradation. To avoid potential DNA damage, we mutated Nme1Cas9 into d_16A_Nme1Cas9 that lost the nuclease activity of the RuvC domain but retained the active HNH domain, able to act on the target DNA strand and, therefore, on the corresponding transcript. Our results showed that d_16A_Nme1Cas9 is a functional RNase *in vivo,* although with moderate activity since it provoked a fluorescence reduction from 21% to 32%. Interestingly, d_16A_Nme1Cas9 works in a PAM-independent way nor demands helper PAMmer molecules. LwaCas13a and RfxCas13d appeared substantially unfunctional in *S. cerevisiae*, though they were shown to perform well in mammalian cells. To the best of our knowledge, this is the first report about the working *in vivo* of a variant of Nme1Cas9 as an RNase and the issues connected with the usage of Cas13 proteins in *S. cerevisiae*.

## Introduction

The clustered regularly interspaced short palindromic repeats (CRISPR)–CRISPR-associated (Cas) protein systems naturally exist in prokaryotes. They are RNA-mediated defense mechanisms to protect bacteria and archaea from invading nucleic acids (Barrangou et al., 2007). CRISPR–Cas systems are classified into two classes (1 and 2). Class 2 CRISPR–Cas require a single effector protein to make a complex with the CRISPR RNA (crRNA) and cleave the target DNA or RNA (Koonin et al., 2017; Makarova et al., 2020). Moreover, class 2 is divided into three types (II, V, and VI), whose representative effector proteins are Cas9, Cas12, and Cas13, respectively. Both Cas9 and Cas12 perform a double-stranded break (DSB) on DNA (Doudna and Charpentier, 2014; Hsu et al., 2014; Zetsche et al., 2015), whereas Cas13 targets and cleaves single-stranded RNA (ssRNA) exclusively (Abudayyeh et al., 2016; Abudayyeh et al., 2017; O'Connell, 2019). The discovery of CRISPR–Cas has greatly promoted the development of genetic engineering and biotechnology (Pickar-Oliver and Gersbach, 2019).

Type II CRISPR–Cas systems are further divided into three subtypes (A, B, and C) (Makarova et al., 2015; Koonin et al., 2017; Makarova et al., 2020). Cas9 protein is an RNA-guided endonuclease that embodies two nuclease domains: HNH and RuvC. Once the trans-activating CRISPR RNA (tracrRNA) and crRNA form a dual-RNA molecules (i.e., tracrRNA:crRNA) and assemble with Cas9 into a ribonucleoprotein, Cas9 is able to recognize a protospacer-adjacent motif (PAM) and bind the target DNA. Then, the two nuclease domains carry out a sequence-specific dsDNA cleavage (Jinek et al., 2012; Jinek et al., 2014; Jiang et al., 2015). In Synthetic Biology applications, the tracrRNA:crRNA duplex is usually replaced by an artificially designed shorter single-guide RNA (sgRNA) that encompasses spacer and direct repeat (DR) (Jinek et al., 2012). Type II-A *Streptococcus pyogenes* Cas9 (SpyCas9) was also harnessed to cleave ssRNA *in vitro* (O’Connell et al., 2014) and track endogenous mRNA in mammalian cells (Nelles et al., 2016) by introducing a PAMmer, i.e., a separate PAM-containing DNA oligonucleotide. Furthermore, both type II-A *Staphylococcus aureus* Cas9 (SauCas9) and type II-C *Campylobacter jejuni* Cas9 (CjeCas9) displayed PAM-independent RNase activity and the capability to cut ssRNA *in vitro* and in bacteria (Dugar et al., 2018; Strutt et al., 2018). Moreover, Strutt et al. demonstrated that, in bacteria, SauCas9 provides protection against invading RNA phages and the nuclease-deficient SauCas9 represses gene expression by binding the target mRNA after making a complex with sgRNA. Finally, type II-C *Neisseria meningitidis* Cas9 (Nme1Cas9) was shown to be able to cleave ssRNA *in vitro* without the need for PAM or PAMmer (Rousseau et al., 2018). However, it is still unknown whether Nme1Cas9 can target and cut transcripts in a PAM-independent way *in vivo*.

The discovery of type VI Cas13 has facilitated the development of a new generation of RNA-based biological tools due to its property of targeting RNA naturally (O'Connell, 2019). Type VI CRISPR-Cas has been subdivided into four subtypes: A–D. A unique feature common to all identified Cas13 proteins is the presence of two distinct HEPN (higher eukaryotes and prokaryotes nucleotide-binding) domains, which function as nuclease domains to cleave the target RNA substrate complementary to the crRNA. Cas13 completely loses its ability to cleave ssRNA if either of the two HEPN domains is mutated (Abudayyeh et al., 2016; East-Seletsky et al., 2016; Shmakov et al., 2017; Makarova et al., 2020). *Leptotrichia wadei* Cas13a (LwaCas13a) has been shown to be functional with no requirement of protospacer-flanking sites (PFSs). It represents the best-performing Cas13a (also known as C2c2) in bacteria and it turned out to reduce effectively, in both human and plant cells, the expression level of either reporter proteins or endogenous genes by targeting their transcripts (Abudayyeh et al., 2017; Gootenberg et al., 2017). Besides, Cas13d from *Ruminococcus flavefaciens* strain XPD3002 (RfxCas13d) showed strong transcript knockdown in human cells (Konermann et al., 2018) and animal (e.g., zebrafish and mouse) embryos (Kushawah et al., 2020) without PFS limitation. Both LwaCas13a and RfxCas13d can target any sequence along a transcript. So far, though, there is no report about their utilization in *Saccharomyces cerevisiae*.

In this work, we first tested the gene editing efficiency, in yeast, of Nme1Cas9 in comparison to that of SpyCas9 (DiCarlo et al., 2013). Then, we harnessed the yeast-codon optimized versions of d_16A_Nme1Cas9 (a partially nuclease-deficient Nme1Cas9 able to cut only the DNA target strand since the D16A mutation inactivated the RuvC domain), LwaCas13a, and RfxCas13d to knockdown the transcripts of reporter genes in *S. cerevisiae*. Our results show that gene editing induced by Nme1Cas9 depends strongly on the target sites and, overall, is not as effective as in SpyCas9 case. Then, we analyzed the action of d_16A_Nme1Cas9 as an RNase. Our results indicate that d_16A_Nme1Cas9 reduces gene expression level by targeting the transcript without the need for PAM and PAMmer. Finally, we utilized LwaCas13a and RfxCas13d to knockdown the transcripts. However, both Cas13 proteins appear unfunctional in *S. cerevisiae*.

## Materials and Methods

### Plasmid Construction

All the plasmids used in this work (see Supplementary Table S1) are based on the pRSII shuttle vector collection (Addgene-35436: pRSII403/HIS3 marker; Addgene-35438: pRSII404/TRP1 marker; Addgene-35440: pRSII405/LEU2 marker; Addgene-35442: pRSII406/URA3 marker; Addgene-35466: pRSII424/TRP1; a gift from Steven Haase) (Chee and Haase, 2012).

Overall, three methods were carried out to construct plasmids: 1) Digestion and ligation. Firstly, the plasmids containing the desired DNA sequences (see below as well) were digested overnight by restriction enzymes such as EcoRI (NEB-R0101S), XbaI (NEB-R0145S), and SalI-HF (NEB-R3138S). Then, the reaction mixture underwent gel electrophoresis and the DNA fragments were purified by AxyPrep DNA Gel Extraction Kit #AP-GX-250. Finally, the purified DNA sequences were ligated by T4 DNA ligase (NEB-M0202S) for 8 h at 16°C. 2) Isothermal assembly (Gibson, 2009). Initially, touchdown PCR was performed to amplify promoters, coding regions, and terminators (see Supplementary Table S2 for their sequences) by using Q5 High-Fidelity DNA Polymerase (NEB-M0491S). Subsequently, the PCR products were isolated and purified as above. The pRSII shuttle vector was digested with SacI-HF (NEB-R3156S) and Acc65I (NEB-R0599S) for 1 h at 37°C to generate a cut-open backbone that was then incubated for 20 min at 65°C to inactivate the two restriction enzymes. Lastly, the purified PCR products were mixed in equimolar amounts with the cut-open backbone and the reaction mixture was left on a PCR machine for 1 h at 50°C. 3) Site-directed mutagenesis. Firstly, we designed two reverse-complementary primers that were 51-nt long. They covered the mutation site and contained the desired mutated sequence (3-nt long) in the middle. Then, touchdown PCR was used to amplify the whole plasmid template. Finally, the reaction mixture was digested by DpnI (NEB-R0176S) for 1 h at 37°C to cleave the original plasmid template and keep the new amplified plasmid containing the mutation site.

DEG1t_pCYC1noTATA and Tsynth8.1_pCYC1noTATA are synthetic promoters constructed in our lab (Song et al., 2016), whereas pGPD, pTEF2, and pSNR52 are constitutive *S. cerevisiae* promoters (notice that pSNR52 is an RNA polymerase III-dependent promoter).

The construct *EcoRI-pGPD-SpyCas9_NLS-ADH1t-XbaI*, derived from Addgene-67638, was placed into the pRSII406 shuttle vector *via* digestion and ligation. Both *XbaI-yo_NLS_Nme1Cas9-SalI* and *XbaI-yo_NLS_d*_*16A*_ *Nme1Cas9-SalI* constructs were generated by site-directed mutagenesis on *yo_NLS_dNme1Cas9,* the yeast-codon optimized version of *dNme1Cas9* (mutations: D16A and H588A), synthesized by GENEWIZ Inc., Suzhou, China. They were placed into the previously constructed acceptor vector *pRSII406-pGPD-ATG-XbaI-SalI-GGTGGA-TAA-CYC1t* by digestion and ligation. *pRSII406-ATG-NLS-GS-HIStag-GS-yo_LwaCas13a-GS-NLS-TAA-CYC1t*, *pRSII406-pGPD-ATG-NLS-GS-HIStag-GS-yo_RfxCas13d-GS-NLS-TAA-CYC1t,* and *pRSII403-(pGDP/pADH1)-ATG-yo_RspWYL1-HAtag-NLS-GS-NLS-TAA-CYC1t* were constructed *via* Gibson assembly. Each yeast-codon optimized Cas13 and WYL1 were synthesized by GENEWIZ Inc., Suzhou, China.

The sgRNA expression cassettes were all build by touchdown PCR and Gibson assembly. They were placed into two kinds of yeast shuttle vectors to achieve different expression levels: the integrative pRSII404/405, which are generally inserted in a single copy into the yeast genome, and the episomal pRSII424 that is present in multiple copies after yeast transformation. The non-target sgRNA in gene editing assay contained a spacer sequence that could not match with any sequence in yeast cells (see Supplementary Table S3 for sgRNA sequences and target positions).

*E. coli*-competent cells (the strain DH5α, Life Technology 18263−012) were used for recombinant plasmid screening and long-term storage (glycerol stocks). The sequences of all the newly constructed plasmids were confirmed by Sanger sequencing at GENEWIZ Inc., Suzhou, China. The oligonucleotides used for PCR and sequencing were synthesized at the same company.

### Yeast Transformation

The parent yeast strain (bYMM584) in this study was *S. cerevisiae* CEN.PK2−1C (MATa; his3D1; leu2-3_112; ura3-52; trp1-289; MAL2-8c; SUC2)—Euroscarf-30000A, Johann Wolfgang Goethe University, Frankfurt, Germany. Yeast cells were transformed either with 5 μg of integrative plasmids—after linearization with a proper restriction enzyme at the auxotrophic marker—or 2 μg of intact episomal plasmids. The PEG/LiAc protocol was followed (Gietz and Woods, 2002). As for gene editing and homology-directed repair, sgRNA was transformed together with 100 ng of donor DNA (see Supplementary Table S2 for donor DNA sequences). Transformants were spread and grown on appropriate synthetic selective medium plates (2% glucose, 2% agar) for 2 days at 30°C. Except for pRSII424-containing yeast strains, which were cultivated on synthetic selective medium all along, the other correct transformants were grown on YPD (yeast extract peptone dextrose) plates (2% glucose, 2% agar) and stored in YPD solution (2% glucose) as 15% glycerol stocks. All engineered yeast strains are listed in Supplementary Table S4.

### Fluorescence Measurement

To detect the fluorescence of each strain, yeast cells were cultured in either SDC (synthetic defined complete medium, 2% glucose) or SD-TRP (synthetic defined medium without tryptophan, 2% glucose, only for the cells transformed with pRSII424-based plasmids) for 20 h. Depending on different experiments, two machines were employed in this work: a flow cytometer and a microplate reader.

The flow cytometer was used for measurements on SpyCas9/Nme1Cas9-mediated gene editing, since gene editing efficiency can vary considerably within a cell population. Therefore, precise analysis at single-cell level appeared the most appropriate. LwaCas13a/RfxCas13d-guided gene repression was estimated *via* FACS (fluorescence activated cell sorting) experiments as well. BD FACSVerse was used to detect fluorescence. The setup for measurements on yEBFP2 (yeast enhanced blue fluorescent protein 2) was: violet laser 405 nm, emission filter 448/45 nm, and Pacific Blue channel (Farzadfard et al., 2013). As for yEGFP, we selected: blue laser 488 nm, emission filter 527/32 nm, and GFP channel. The BD FACSVerse setup was checked at the beginning and the end of each experiment by means of fluorescent beads (BD FACSuite CS&T Research Beads—650621) to assure that the experimental conditions were stable and the results from independent experiments were comparable.

The microplate reader was harnessed to monitor yEBFP2 fluorescence in d_16A_Nme1Cas9-mediated gene repression. After culturing the yeast cells, they were harvested into 2 mL microtubes and centrifuged at the maximum speed for 1 min. Then the supernatant was discarded and the cells were resuspended in 500 μL of double distilled water (ddH_2_0). 200 μL of cell resuspension solution were poured into single wells of a 96-well flat bottom black microplate to measure fluorescence intensity (excitation wavelength—383 nm; emission wavelength—448 nm) (Ai et al., 2007). 20 μL of cell resuspension solution were diluted into 180 μL of ddH_2_0 (1:10 dilution) before measuring the absorbance (optical density) at 600 nm (OD_600_). Finally, the average fluorescence over a cell population was calculated as:

$$Mean\;Fluorescence=\frac{Fluorescence\;Intensity}{Absorbance_{600nm}\times 10}$$

## Results

### SpyCas9/Nme1Cas9-Mediated Gene Editing in *S. cerevisiae*

**FIGURE 1:**
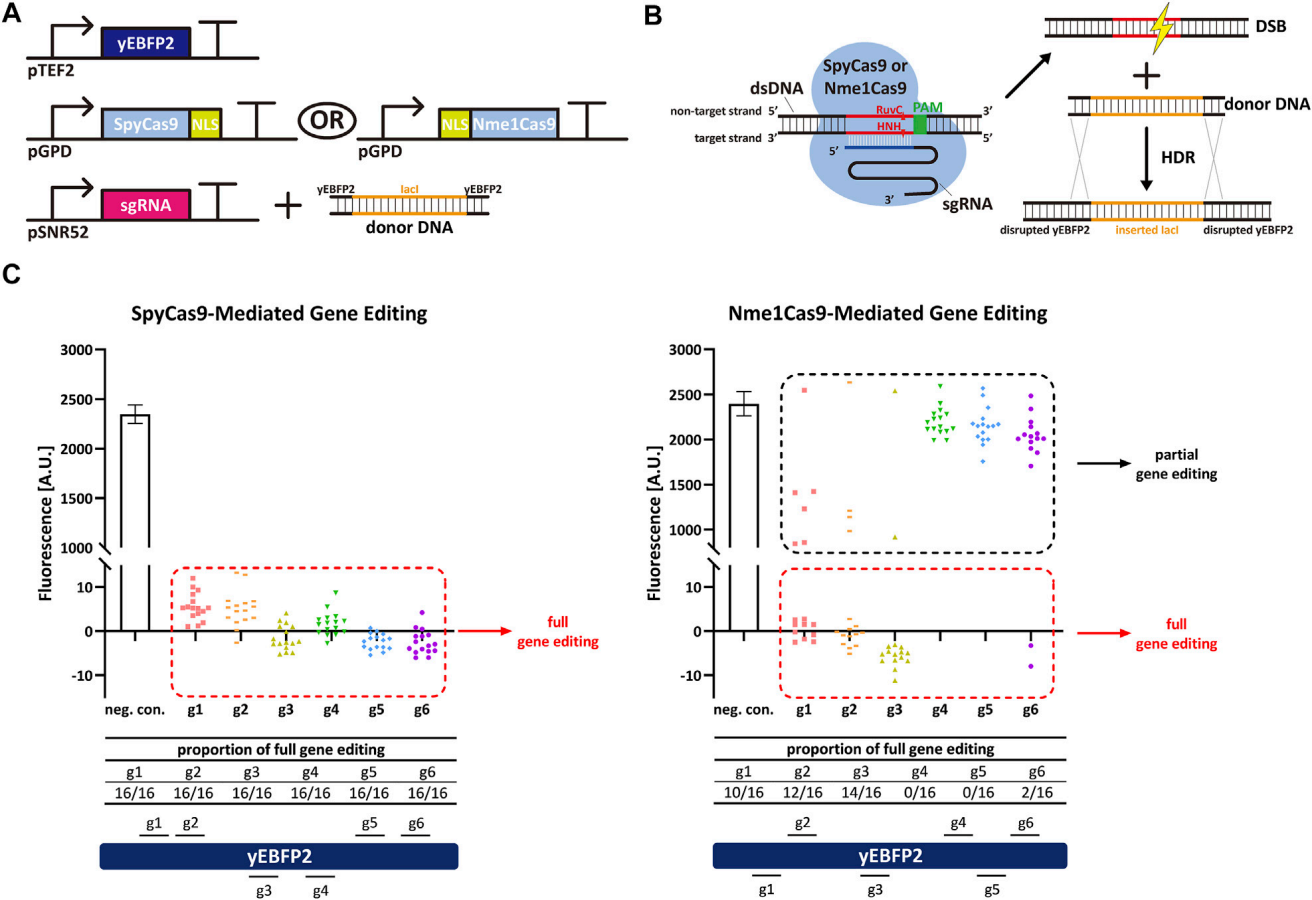
Spy/Nme1Cas9-mediated gene editing in *S. cerevisiae*. **(A)** Schematic representation of the transcription units integrated into the yeast genome to carry out gene editing together with the donor DNA delivered to the cells. Black right-turn arrows are promoters; T-shape symbols represent terminators. In the donor DNA, the orange part comes from the coding sequence of bacterial *lacI*, whereas the black parts are sequences of *yEBFP2* serving as the homologous arms. **(B)** Mechanism of Cas9-mediated gene editing and homology directed repair (HDR). **(C)** Fluorescence levels of Spy/Nme1Cas9-mediated gene editing. The first bar is the negative control (a non-targeted sgRNA was expressed) whose fluorescence level is the mean value from three independent experiments, and the error bar is the standard deviation of the mean. The other columns represented test groups. Each test group contains sixteen points, and every point corresponds to the fluorescence of a single transformant. gi, i = 1, … , 6 stands for sgRNAi. Red dashed frames highlight the strains that were fully edited by Cas9. Their fluorescence was completely repressed as indicated by the presence of a single-narrow peak in the fluorescence histogram (see Supplementary Figure S1A). Black dashed frames mark the strains that were only partially edited by Cas9, i.e., their fluorescence histogram showed two peaks (see Supplementary Figure S1B,C). Segments above (below) the yEBFP2 gene point out that the corresponding sgRNAs targeted the sense (antisense) strand of yEBFP2.

Firstly, we harnessed yeast-codon optimized versions of SpyCas9 and Nme1Cas9 to carry out gene editing and compare their performance and efficiency in *S. cerevisiae*. Both Cas9 proteins were fused to a nuclear localization sequence (NLS) and expressed in high amount by the very strong *GPD* promoter. The yeast enhanced blue fluorescent protein 2 (yEBFP2) (Ai et al., 2007; Farzadfard et al., 2013) was chosen as a reporter and expressed under the moderately strong *TEF2* promoter. *yEBFP2* was the target for both SpyCas9 and Nme1Cas9. As the two Cas9 homologs recognize incompatible PAMs (NGG—or NAG—by SpyCas9 and NNNNGATT by Nme1Cas9), and the natural lengths of the sgRNA spacer are also different (20 nt for SpyCas9 and 24 nt for Nme1Cas9), we selected, for each Cas9, six targets along *yEBFP2* (see Supplementary Table S3). After successively transforming yeast cells with the transcription units (TUs) encoding for yEBFP2, Cas9, and the sgRNA—the latter together with the donor DNA (a portion of the bacterial *lacI* sequence flanked at both ends by partial sequences of *yEBFP2* as the homologous arms, see Figure 1A)—the DSB caused by Cas9 on *yEBFP2* could be repaired *via* homologous recombination. Thus, the *lacI-*derived sequences were inserted into the coding sequence of yEBFP2, which prevented the expression of functional yEBFP2 (see Figure 1B). We randomly selected sixteen colonies for the FACS experiments. As shown in Figure 1C, SpyCas9 showed much higher efficiency and better performance than Nme1Cas9. SpyCas9 fully edited *yEBFP2* and turned off its expression at all six target positions in each colony. In contrast, Nme1Cas9 gave, as a highest editing efficiency, the complete knockdown of 14 out of 16 colonies in combination with sgRNA3. Furthermore, none of the colonies edited at position 4 and 5 resulted in a full yEBFP2 shut down and only a partial gene editing was observed (see also the Fluorescence-Cell Count histogram in Supplementary Figure S1). Therefore, we concluded that Nme1Cas9 works in *S. cerevisiae*, although its gene editing activity strongly depends on the target sites, in agreement with previous results on genome editing in human cells (Amrani et al., 2018). Moreover, Nme1Cas9 is not as effective as SpyCas9. This is also consistent with results in human cells (Lee et al., 2016; Wang et al., 2018).

### d_16A_Nme1Cas9-Mediated Gene Repression by Targeting the Transcripts in *S. cerevisiae*

**FIGURE 2:**
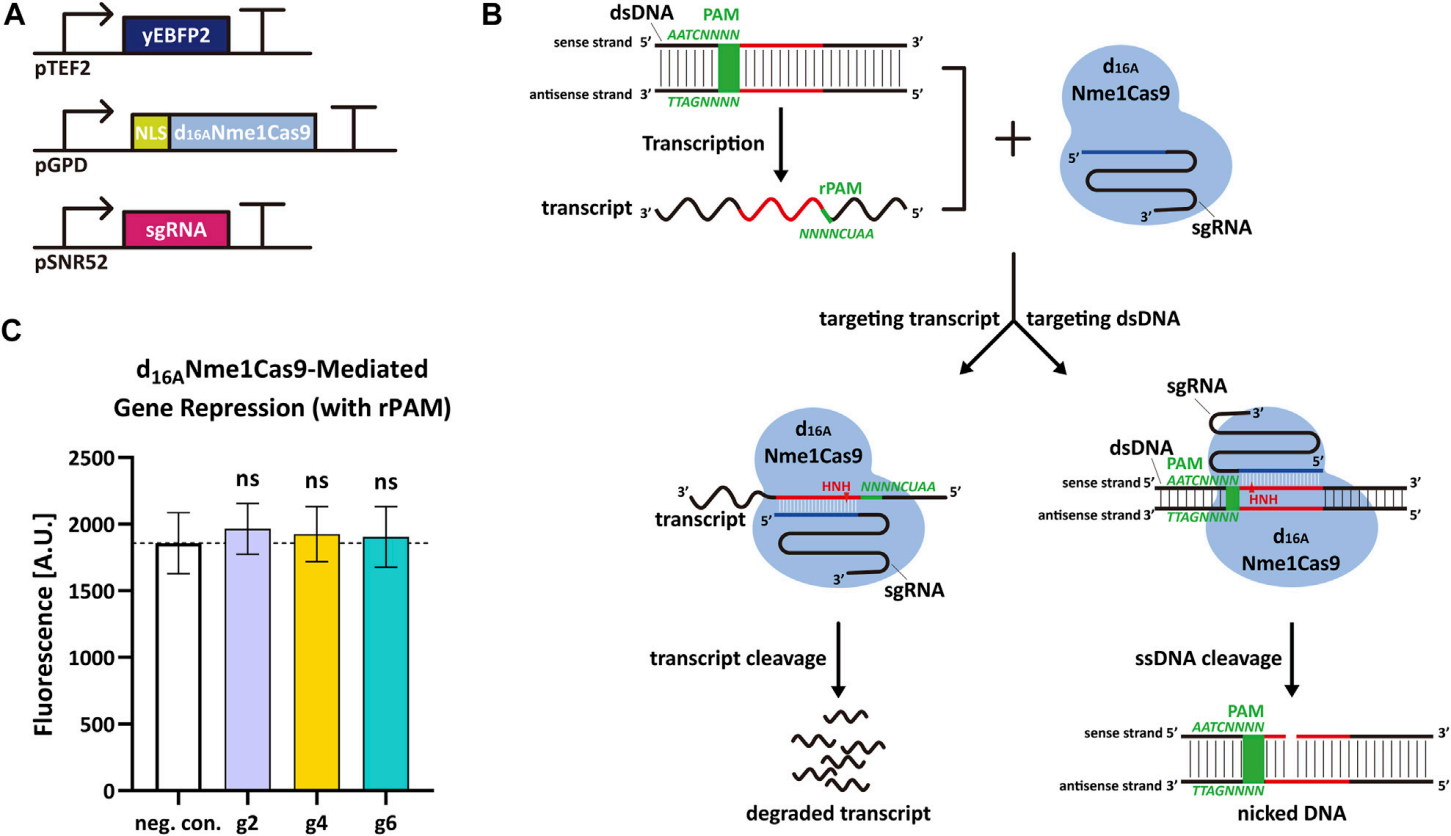
d_16A_Nme1Cas9-mediated mRNA degradation in *S. cerevisiae*. **(A)** Schematic representation of the transcription units integrated into the yeast genome to perform *yEBF2* transcript degradation. **(B)** Mechanism diagram. d_16A_Nme1Cas9 binds the dsDNA substrate upstream of 5′-NNNNGATT-3′ PAM sequence and DNA transcripts downstream of 5′-AAUCNNNN-3′ rPAM sequence. Due to the active HNH nuclease domain and an unfunctional RuvC domain, d_16A_Nme1Cas9 is capable of cutting the sense strand of the dsDNA (acting as a DNA nickase) as well as its transcript. **(C)** Fluorescence level of d_16A_Nme1Cas9-mediated gene repression. “neg. con.,” stands for negative control (no sgRNA was expressed). g2, g4, and g6 are the same sgRNAs as in Figure 1. The “ns” indicates no significant difference between the corresponding test strains and the negative control (two-tailed Welch’s t-test). Each fluorescence level represents the mean value from 12 measurements. Error bars are the standard deviation of the mean.

Nme1Cas9 is able to cleave ssRNA containing rPAM (5′-AAUCNNNN-3′, the PAM equivalent in RNA molecule) *in vitro* (Rousseau et al., 2018). Thus, we decided to verify if Nme1Cas9 could work as an RNase and knock down the expression of *yEBFP2* without permanent DNA damage in *S. cerevisiae*. We started from some preliminary tests based on the above gene editing experiments. To avoid the DSB generated by fully-active Nme1Cas9, we silenced its RuvC nuclease domain (which cuts the non-target dsDNA strand) *via* the D16A mutation (Zhang et al., 2015; Rousseau et al., 2018). In this way, we engineered a new version of Nme1Cas9, termed d_16A_Nme1Cas9, that owned only the HNH nuclease activity. We hypothesized that, upon combination with an sgRNA complementary to a segment of the sense strand of the DNA, d_16A_Nme1Cas9 could bind both the dsDNA and its transcripts after recognizing PAM and rPAM, respectively. The active HNH domain would only nick the DNA on the sense strand (no DSB) and cleave the transcripts. Hence, d_16A_Nme1Cas9 could lower gene expression by triggering mRNA degradation (Figures 2A,B). In this initial test, we expressed yEBFP2, NLS-d_16A_Nme1Cas9, and sgRNA2, sgRNA4 or sgRNA6 (they are reverse complement to segments of *yEBFP2* sense strand) to see if there was any reduction in fluorescence. However, we did not observe any gene repression (Figure 2C). Plausible explanations for our negative results are: 1) the *TEF2* promoter drove the synthesis of too much mRNA that could not be fully cleaved by d_16A_Nme1Cas9; 2) d_16A_Nme1Cas9 was localized into the nucleus because it was fused to an NLS. We chose this configuration because previous works reported that the mRNA knockdown by the RNase Cas13 was enhanced by fusing it to two NLSs (Abudayyeh et al., 2017; Konermann et al., 2018). However, Cas13 binds RNA exclusively, whereas Nme1Cas9:sgRNA is a DNA-targeting ribonucleoprotein. Therefore, d_16A_Nme1Cas9 has probably a much higher affinity to the target DNA rather than to its transcripts in nucleus. Furthermore, the NLS might preclude the processing of the mature mRNA in the cytoplasm. According to these considerations, we made further attempts to improve our results.

**FIGURE 3:**
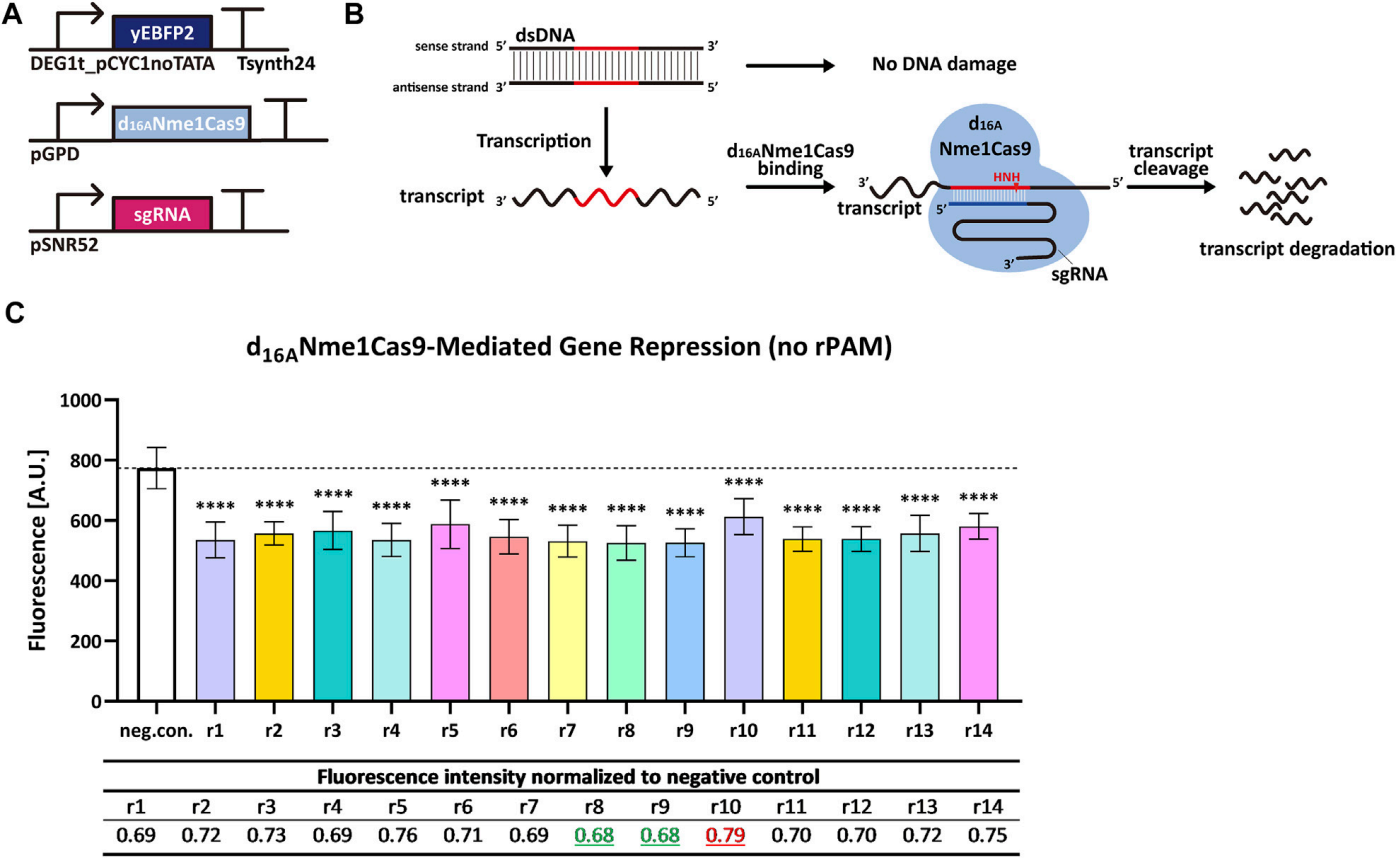
d_16A_Nme1Cas9-mediated gene repression in *S. cerevisiae* by targeting transcripts without rPAM. **(A)** Schematic representation of the transcription units integrated into the yeast genome for the new experiments. **(B)** Mechanism diagram. Because the sgRNA has no relation with PAM, d_16A_Nme1Cas9 cannot target the DNA. However, d_16A_Nme1Cas9 can bind the mRNA in an rPAM-independent way and trigger its degradation. **(C)** Fluorescence level of d_16A_Nme1Cas9-mediated gene repression. “neg. con.” is the negative control (no sgRNA was expressed). ri, i = 1, … , 14 stands for sgRNAri that guides d_16A_Nme1Cas9 to the *yEBFP2* transcript. In particular, sgRNAr14 was designed to bind the poly(A) site in the 3′ UTR (untranslated region) from the synthetic terminator Tsynth24 (Curran et al., 2015). The black dashed line marked the fluorescence level of the negative control. ^∗^^∗^^∗^^∗^: *p-value* < 0.0001, indicates a statistically significant difference between the corresponding test strain and the negative control (two-tailed Welch’s t-test). Each fluorescence level represents the mean value from 12 measurements. Error bars are the standard deviation of the mean. The table below the bar plot reports the normalized fluorescence levels with respect to the negative control: sgRNAr8/r9 gave the biggest reduction in fluorescence (32%), whereas sgRNAr10 the smallest one (21%).

We replaced the *TEF2* promoter with a relatively weak synthetic promoter—DEG1t_pCYC1noTATA—to produce yEBFP2. Besides, we removed the NLS from d_16A_Nme1Cas9. A triple mutation of PAM has been reported to abrogate the ability of Nme1Cas9 to induce DSB on the DNA (Zhang et al., 2015). In contrast, rPAM-mutated RNA was cut, *in vitro*, as effectively as the RNA carrying the correct rPAM (Rousseau et al., 2018). This suggests that Nme1Cas9, in complex with a properly designed sgRNA, can work on ssRNA in a PAM-independent manner without any interaction with the DNA. We designed a group of new 14 sgRNAs (reverse complement to *yEBFP2* sense strand and named from sgRNAr1 to sgRNAr14) independent of PAM, and supposed that d_16A_Nme1Cas9 would target mRNA exclusively upon binding each of these new 14 sgRNAri. Experimental results confirmed our hypothesis since every sgRNAri induced a decrease in fluorescence, ranging from 21% to 32% (see Figure 3).

### LwaCas13a/RfxCas13d-Mediated Gene Repression

**FIGURE 4:**
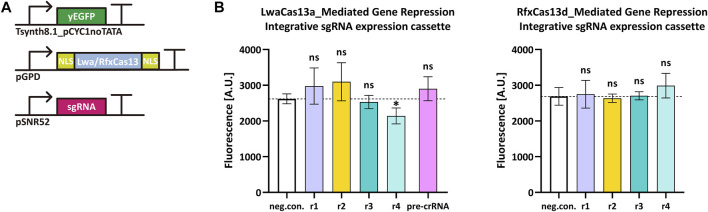
LwaCas13a/RfxCas13d-mediated gene repression in *S. cerevisiae*. **(A)** Schematic representation of the transcription units transformed into the yeast genome. **(B)** Fluorescence level of LwaCas13a/RfxCas13d-mediated gene repression when the sgRNA expression cassette was inserted into an integrative plasmid. “neg. con.” is the negative control (no sgRNA was expressed). ri, i = 1, … ,4 stands for sgRNAri. The black dashed line marks the fluorescence level of the negative control. ^∗^: *p-value* < 0.05, indicates a statistically significant difference between the corresponding test strain and the negative control; “ns” means no significant difference between the corresponding test strains and the negative control (two-tailed Welch’s t-test). Each fluorescence level represents the mean value from three independent measurements with a flow cytometer. Error bars are the standard deviation of the mean.

Cas13 proteins target RNA only upon binding a crRNA molecule. Type VI-A LwaCas13a and type VI-D RfxCas13d showed high efficiency in mammalian cells (Abudayyeh et al., 2017; Konermann et al., 2018) and a general ease-of-use since they bound mRNA without any constraints such as PFSs. We tested how these two Cas13 proteins worked in *S. cerevisiae* by targeting the yeast enhanced green fluorescent protein (*yEGFP*) (Sheff and Thorn, 2004) mRNA. We chose a rather weak synthetic promoter—Tsynth8.1_pCYC1noTATA (Song et al., 2016)—to express yEGFP. Two NLSs were separately placed at the N- and C-terminus of both LwaCas13a and RfxCas13d since they guaranteed best performance in mammalian cells (Abudayyeh et al., 2017; Konermann et al., 2018). We designed four sgRNAr that targeted different positions along the *yEGFP* transcript. Initially, we placed the sgRNAr expression cassette into an integrative shuttle vector, as in the above experiments with Nme1Cas9 and derivatives (Figure 4A). However, only LwaCas13a:sgRNAr4 showed a modest reduction in fluorescence (18.2%) that was significantly different from the negative control, whereas RfxCas13d did not work in a complex with any of the four sgRNAri (Figure 4B). Since Cas13 proteins process their corresponding pre-crRNA to generate mature crRNA (East-Seletsky et al., 2016; East-Seletsky et al., 2017), we also used, together with LwaCas13a, a pre-crRNA that was composed of three different direct repeat-spacer (DR-spacer) sequences, each in a double copy i.e., the first three DR-spacers were the same as the last three. In this way, we increased the amount of sgRNAr molecules in cells and simultaneously targeted three sites on the *yEGFP* transcript. However, this solution turned out to be ineffective too (see Figure 4).

We tried to further enhance sgRNAr concentration by placing the sgRNAr expression cassette inside an episomal plasmid. In principle, there should be 10–40 copies of the plasmid inside the cells after transformation. This expedient, though, led to no improvement either (see Supplementary Figure S2).

We turned to other strategies to try to make the CRISPR–Cas13 system work. Since RfxCas13d was reported to outperform LwaCas13 in mammalian cells (Konermann et al., 2018) and work efficiently also in animal embryos (Kushawah et al., 2020), we focused on the sole RfxCas13d for our next tests. Firstly, we changed the DR sequence on our sgRNAr from the one in (Konermann et al., 2018) (“DR (UrCas13d),” 5′-AAC​CCC​TAC​CAA​CTG​GTC​GGG​GTT​TGA​AAC-3′), to the more general version (“standard DR,” 5′-AAC​CCT​ACC​AAC​TGG​TCG​GGG​TTT​GAA​AC-3′) and an optimized one (“optimal DR,” 5′-TAC​CCT​ACC​AAC​TGG​TCG​GGG​TTT​GAA​AC-3′) reported in (Wessels et al., 2020). We used both standard and optimal DR together with sgRNAr1 and sgRNAr2 only. Moreover, Yan et al. pointed out that the WYL1 protein enhanced the RNase activity of type VI-D CRISPR–Cas13 systems (Yan et al., 2018). In particular, WYL1 from *Ruminococcus* sp. N15.MGS-57 (RspWYL1) increased the ssRNA cleavage efficiency of both RspCas13d and EsCas13d (from *Eubacterium siraeum*). Therefore, we thought that RspWYL1 might activate RfxCas13d-mediated transcript degradation in yeast. Consequently, we added RspWYL1 in the circuit drawn in Figure 4A. However, neither the new DR sequences nor the expression of RspWYL1 altered our previous results (see Supplementary Figure S3A,B), even when combining them (new DR plus RspWYL1, see Supplementary Figure S3C. Hence, we had to conclude that our systems based on LwaCas13a/RfxCas13d for mRNA degradation in *S. cerevisiae* were non-functional.

## Discussion

Cas9:sgRNA is an RNA-guided DNA-targeting ribonucleoprotein that has become a programmable genome engineering tool, as well as a template for building new transcription factors (upon silencing its nuclease activity) to up- or down-regulate gene expression (Sander and Joung, 2014; Marchisio and Huang, 2017; Pickar-Oliver and Gersbach, 2019). Interestingly, some studies showed that several Cas9 homologs (e.g., SpyCas9, SauCas9, CjeCas9, and Nme1Cas9) naturally possess RNase activity i.e., they also recognize, target, and cleave ssRNA *in vitro* and, in some cases, in bacteria (O’Connell et al., 2014; Dugar et al., 2018; Rousseau et al., 2018; Strutt et al., 2018). Therefore, it is worth investigating the possibility to use Cas9 homologs as tools to modulate mRNA degradation in eukaryotes—perhaps without the need for PAM or PAMmer molecules.

Type II-C Nme1Cas9 has been proven to be functional for ssRNA cleavage *in vitro* (Rousseau et al., 2018), but there are no reports about its usage *in vivo*. Here, we investigated the working and performance of Nme1Cas9 in *S. cerevisiae*. Our results show that Nme1Cas9 carries out gene editing in *S. cerevisiae*, though in a highly target-dependent way and with lower efficiency than SpyCas9. Importantly, we engineered a RuvC-silenced version of this protein, termed d_16A_Nme1Cas9, that proved to be able to trigger the degradation of the transcript of a reporter gene—*yEBFP2*—with a consequent decrease in fluorescence ranging from 21% to 32%. Moreover, d_16A_Nme1Cas9 worked independently of PAM sequences and PAMmer molecules [the latter are needed, for instance, by SpyCas9 for RNA-targeting applications such as *in vivo* ssRNA cleavage (O’Connell et al., 2014) and real-time tracking of mRNA in living cells (Nelles et al., 2016)]. Unfortunately, d_16A_Nme1Cas9 gave only moderate performance.

We also studied two Cas13 homologs, LwaCas13a and RfxCas13d, as a means to modulate mRNA degradation in *S. cerevisiae*. Since Cas13 targets RNA only and proved to work in mammalian cells, we expected that it was functional in yeast too. However, only one out of 35 tests returned a positive result. So far, we were not able to find a working configuration even though we changed several variables such as the sgRNA amount, the target location on the mRNA, the sequence of the DR, and the expression of the WYL1 accessory protein. It should be noted that a different Cas13 homolog, i.e., LshCas13a (from *Leptotrichia Shahii*) was reported to be able to knockdown transcripts in the yeast *Schizosaccharomyces pombe*, though with different efficiency on different genes (Jing et al., 2018).

In short, our results show that d_16A_Nme1Cas9 is capable of transcript knockdown *in vivo* in a PAM/PAMmer-independent fashion, though with moderate effectiveness. In contrast, LwaCas13a and RfxCas13d appeared unfunctional in *S. cerevisiae*. Both systems, nevertheless, require a deeper study, on one hand to understand the full potency of Nme1Cas9-based RNA editing, on the other hand to find what hinders the working of Cas13 proteins in *S. cerevisiae*. Improvements in both directions would be extremely useful to design synthetic gene circuits that make use of mRNA degradation as a control of translation.

## Data Availability

The datasets presented in this study have been deposited at https://www.jianguoyun.com/p/DYO6PXQQtIDFChjg4bsEIAA.
