# Supplementary material for: A Mutated Nme1Cas9 Is a Functional Alternative RNase to Both LwaCas13a and RfxCas13d in the Yeast S. cerevisiae
Source: Front Bioeng Biotechnol. 2022 Jun 2;10:922949. doi: 10.3389/fbioe.2022.922949 (PMC9201564; doi:10.3389/fbioe.2022.922949)
Supplement: Supplementary file 1 [file DataSheet1.pdf]

Supplementary Material

A mutated Nme1Cas9 is a functional alternative RNase to both LwaCas13a and RfxCas13d in the yeast *S. cerevisiae*

Yadan Zhang[1], Huanhuan Ge[1], and Mario Andrea Marchisio[1*]

[1] School of Pharmaceutical Science and Technology, Tianjin University, 92 Weijin Road, 300072-Tianjin, China
[*] corresponding author. Email: mario@tju.edu.cn or mamarchisio@yahoo.com

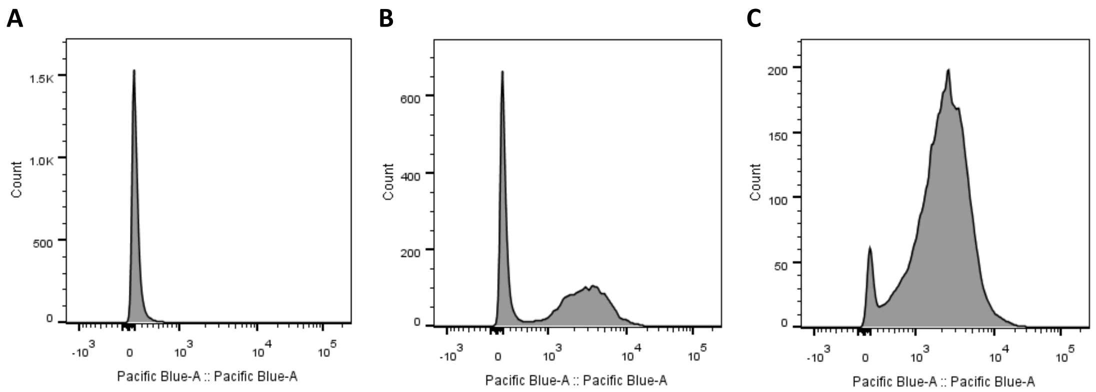

Figure S1. Fluorescence-Cell count histogram of FACS experiments. (A) Full gene editing; (B) partial gene editing with relatively low fluorescence; (C) partial gene editing with relatively high fluorescence.

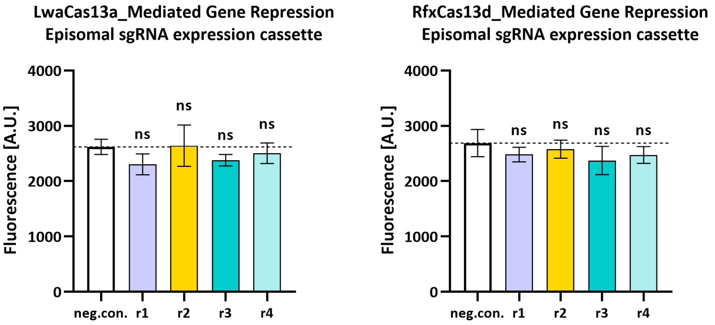

Figure S2. Fluorescence level of LwaCas13a/RfxCas13d-mediated gene repression when the sgRNA expression cassette was inserted into an episomal shuttle vector. “neg. con.” represents the negative control (no sgRNA was expressed). ri, i=1,…,4 stands for sgRNAri. The black dashed line marks the fluorescence level of negative control. “ns” indicates no significant difference between the corresponding test strains and the negative control (two-tailed Welch’s t-test). Each fluorescence level represents the mean value from three independent measurements at the FACS machine. Error bars are the standard deviation of the mean.

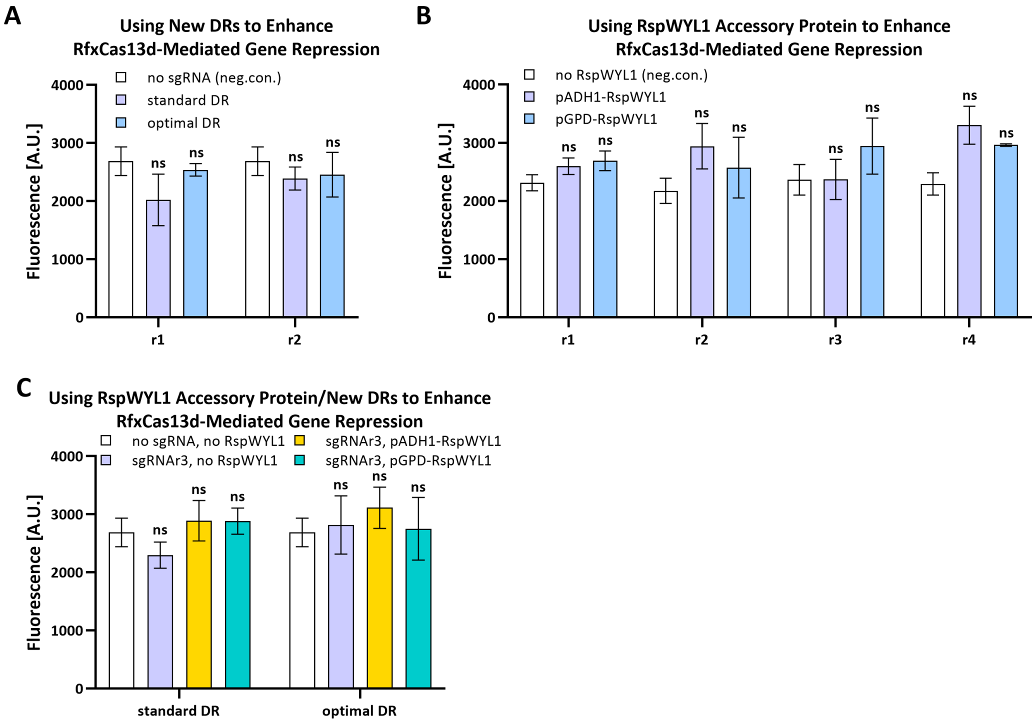

Figure S3. Fluorescence level of RfxCas13d-mediated gene repression. (A) New DRs replaced the one initially used. (B) RspWYL1 is added to the original circuit and expressed under either pGPD (a very strong promoter) or pADH1 (a moderately strong promoter). (C) The combination of new DRs and RspWYL1. ri, i=1,…,4, stands for sgRNAri. "ns" indicates no significant difference between the corresponding test strains and the negative control (two-tailed Welch's t-test). Each fluorescence level represents the mean value from three independent measurements at the FACS machine. Error bars are the standard deviation of the mean.

Table S1. Plasmids constructed in this work.

NAME	CONTENT
pMM1309	pRSII404-pTEF2-yEBFP2-CYC1t_atc
pMM1379	pRSII404-DEG1t_pCYC1noTATA-yEBFP2-Tsynth24
pMM1303	pRSII406-EcoRI-pGPD-SpyCas9_NLS-ADH1t-XbaI
pMM1321	pRSII406-pGPD-ATG-XbaI-yo_NLS_d_{16A}Nme1Cas9-SalI-GGTGGA-TAA(STOP)-CYC1t
pMM1322	pRSII406-pGPD-ATG-XbaI-yo_NLS_Nme1Cas9-SalI-GGTGGA-TAA(STOP)-CYC1t
pMM1424	pRSII406-pGPD-ATG-XbaI-yo_d_{16A}Nme1Cas9-GS-HAtag-SalI-TAA-CYC1t
pMM1325	pRSII405-pSNR52-sgRNA1_yEBFP2_DR(Nme1Cas9)-SUP4t
pMM1349	pRSII405-pSNR52-sgRNA2_yEBFP2_DR(Nme1Cas9)-SUP4t
pMM1326	pRSII405-pSNR52-sgRNA3_yEBFP2_DR(Nme1Cas9)-SUP4t
pMM1350	pRSII405-pSNR52-sgRNA4_yEBFP2_DR(Nme1Cas9)-SUP4t
pMM1327	pRSII405-pSNR52-sgRNA5_yEBFP2_DR(Nme1Cas9)-SUP4t
pMM1351	pRSII405-pSNR52-sgRNA6_yEBFP2_DR(Nme1Cas9)-SUP4t
pMM1381	pRSII405-pSNR52-random sequence(24nt)_DR(Nme1Cas9)-SUP4t
pMM1328	pRSII405-pSNR52-sgRNA1_yEBFP2_DR(SpyCas9)-SUP4t
pMM1352	pRSII405-pSNR52-sgRNA2_yEBFP2_DR(SpyCas9)-SUP4t
pMM1329	pRSII405-pSNR52-sgRNA3_yEBFP2_DR(SpyCas9)-SUP4t
pMM1353	pRSII405-pSNR52-sgRNA4_yEBFP2_DR(SpyCas9)-SUP4t
pMM1330	pRSII405-pSNR52-sgRNA5_yEBFP2_DR(SpyCas9)-SUP4t
pMM1354	pRSII405-pSNR52-sgRNA6_yEBFP2_DR(SpyCas9)-SUP4t
pMM1402	pRSII405-pSNR52-random sequence(20nt)_DR(SpyCas9)-SUP4t
pMM1445	pRSII405-pSNR52-sgRNAr1_yEBFP2_DR(Nme1Cas9)-SUP4t
pMM1428	pRSII405-pSNR52-sgRNAr2_yEBFP2_DR(Nme1Cas9)-SUP4t
pMM1429	pRSII405-pSNR52-sgRNAr3_yEBFP2_DR(Nme1Cas9)-SUP4t
pMM1453	pRSII405-pSNR52-sgRNAr4_yEBFP2_DR(Nme1Cas9)-SUP4t
pMM1426	pRSII405-pSNR52-sgRNAr5_yEBFP2_DR(Nme1Cas9)-SUP4t
pMM1446	pRSII405-pSNR52-sgRNAr6_yEBFP2_DR(Nme1Cas9)-SUP4t
pMM1447	pRSII405-pSNR52-sgRNAr7_yEBFP2_DR(Nme1Cas9)-SUP4t
pMM1430	pRSII405-pSNR52-sgRNAr8_yEBFP2_DR(Nme1Cas9)-SUP4t
pMM1448	pRSII405-pSNR52-sgRNAr9_yEBFP2_DR(Nme1Cas9)-SUP4t
pMM1427	pRSII405-pSNR52-sgRNAr10_yEBFP2_DR(Nme1Cas9)-SUP4t
pMM1454	pRSII405-pSNR52-sgRNAr11_yEBFP2_DR(Nme1Cas9)-SUP4t

pMM1455	pRSII405-pSNR52-sgRNAr12_yEBFP2_DR(Nme1Cas9)-SUP4t
pMM1431	pRSII405-pSNR52-sgRNAr13_yEBFP2_DR(Nme1Cas9)-SUP4t
pMM1380	pRSII405-pSNR52-sgRNAr14_yEBFP2_DR(Nme1Cas9)-SUP4t
pMM728	pRSII405-Tsynth8.1_pCYC1noTATA-yEGFPgg-CYC1t_atc
pMM813	pRSII406-pGPD-ATG-NLS-GS-HIStag-GS-yo_LwaCas13a-GS-NLS-TAA-CYC1t
pMM843	pRSII406-pGPD-ATG-NLS-GS-HIStag-GS-yo_RfxCas13d-GS-NLS-TAA-CYC1t
pMM805	pRSII404-pSNR52-sgRNAr1_yEGFPgg_DR(LwaCas13a)-SUP4t
pMM806	pRSII404-pSNR52-sgRNAr2_yEGFPgg_DR(LwaCas13a)-SUP4t
pMM933	pRSII404-pSNR52-sgRNAr3_yEGFPgg_DR(LwaCas13a)-SUP4t
pMM888	pRSII404-pSNR52-sgRNAr4_yEGFPgg_DR(LwaCas13a)-SUP4t
pMM673	pRSII404-pSNR52-RL-s(79)-RL-s(116)-RL-s(265)-RL-s(79)-RL-s(116)-RL-s(265)-SUP4t (RL: Repeat LwaCas13a; s: spacer)
pMM792	pRSII424-pSNR52-sgRNAr1_yEGFPgg_DR(LwaCas13a)-SUP4t
pMM793	pRSII424-pSNR52-sgRNAr2_yEGFPgg_DR(LwaCas13a)-SUP4t
pMM934	pRSII424-pSNR52-sgRNAr3_yEGFPgg_DR(LwaCas13a)-SUP4t
pMM889	pRSII424-pSNR52-sgRNAr4_yEGFPgg_DR(LwaCas13a)-SUP4t
pMM839	pRSII404-pSNR52-sgRNAr1_yEGFPgg_DR(UrCas13d)-SUP4t
pMM840	pRSII404-pSNR52-sgRNAr2_yEGFPgg_DR(UrCas13d)-SUP4t
pMM935	pRSII404-pSNR52-sgRNAr3_yEGFPgg_DR(UrCas13d)-SUP4t
pMM890	pRSII404-pSNR52-sgRNAr4_yEGFPgg_DR(UrCas13d)-SUP4t
pMM841	pRSII424-pSNR52-sgRNAr1_yEGFPgg_DR(UrCas13d)-SUP4t
pMM842	pRSII424-pSNR52-sgRNAr2_yEGFPgg_DR(UrCas13d)-SUP4t
pMM936	pRSII424-pSNR52-sgRNAr3_yEGFPgg_DR(UrCas13d)-SUP4t
pMM891	pRSII424-pSNR52-sgRNAr4_yEGFPgg_DR(UrCas13d)-SUP4t
pMM1062	pRSII404-pSNR52-sgRNAr1_yEGFPgg_RfxCas13d(optimal DR)-SUP4t
pMM1064	pRSII404-pSNR52-sgRNAr2_yEGFPgg_RfxCas13d(optimal DR)-SUP4t
pMM995	pRSII404-pSNR52-sgRNAr3_yEGFPgg_RfxCas13d(optimal DR)-SUP4t
pMM1063	pRSII404-pSNR52-sgRNAr1_yEGFPgg_RfxCas13d(standard DR)-SUP4t
pMM1065	pRSII404-pSNR52-sgRNAr2_yEGFPgg_RfxCas13d(standard DR)-SUP4t
pMM997	pRSII404-pSNR52-sgRNAr3_yEGFPgg_RfxCas13d(standard DR)-SUP4t
pMM1022	pRSII403-pADH1-ATG-Rsp_WYL1(with HAtag)-NLS-GS-NLS-TAA-CYC1t_atc
pMM1023	pRSII403-pGPD-ATG-Rsp_WYL1(with HAtag)-NLS-GS-NLS-TAA-CYC1t_atc

Table S2. DNA sequences used in this work.

NAME	SEQUENCE
pTEF2	GCTACCTATATTCCACCATAACATCAATCATGCGGTTGCTGGTGTATTTACCAATAATG<br>TTTAATGTATATATATATATATATATGGGGCCGTATACTTACATATAGTAGATGTCAAGCG<br>TAGGCGCTTCCCCTGCCGGCTGTGAGGGCGCCATAACCAAGGTATCTATAGACCGC<br>CAATCAGCAAACTACCTCCGTACATTCATGTTGCACCCACACATTTATACACCCAGAC<br>CGCGACAAATTACCCATAAGGTTGTTTGTGACGGCGTCGTACAAGAGAACGTGGG<br>AACTTTTTAGGCTCACCAAAAAAGAAAGAAAAAATACGAGTTGCTGACAGAAGCC<br>TCAAGAAAAAAAAAATTCTTCTTCGACTATGCTGGAGGCAGAGATGATCGAGCCGG<br>TAGTTAACTATATATAGCTAAATTGGTTCCATCACCTTCTTTTCTGGTGTCGCTCCTTCT<br>AGTGCTATTTCTGGCTTTTCCTATTTTTTTTTTTCCATTTTTCTTTCTCTCTTTCTAATAT<br>ATAAATTCTCTTGCATTTTCTATTTTTCTCTCTATCTATTCTACTTGTTTATTCCCTTCAA<br>GGTTTTTTTTTAAGGAGTACTTGTTTTTAGAATATACGGTCAACGAACTATAATTAAC<br>TAAAC
pGPD	CAGTTCGAGTTTATCATTATCAATACTGCCATTTCAAAGAATACGTAAATAATTAATAG<br>TAGTGATTTTCCTAACTTTATTTAGTCAAAAAATTAGCCTTTTAATTCTGCTGTAACCC<br>GTACATGCCCAAAATAGGGGGCGGGTTACACAGAATATATAACATCGTAGGTGTCTG<br>GGTGAACAGTTTATTCCTGGCATCCACTAAATATAATGGAGCCCGCTTTTTAAGCTG<br>GCATCCAGAAAAAAAAAGAATCCCAGCACCAAAATATTGTTTTCTTCACCAACCATC<br>AGTTCATAGGTCCATTCTCTTAGCGCAACTACAGAGAACAGGGGCACAAACAGGCA<br>AAAAACGGGCACAACCTCAATGGAGTGATGCAACCTGCCTGGAGTAAATGATGAC<br>ACAAGGCAATTGACCCACGCATGTATCTATCTCATTTTCTTACACCTTCTATTACCTTC<br>TGCTCTCTCTGATTTGGAAAAAGCTGAAAAAAAAGGTTGAAACCAGTTCCCTGAAA<br>TTATTCCCCTACTTGACTAATAAGTATATAAAGACGGTAGGTATTGATTGTAATTCTGT<br>AAATCTATTTCTTAAACTTCTTAAATTCTACTTTTATAGTTAGTCTTTTTTTTAGTTTTA<br>AAACACCAAGAACTTAGTTTCGAATAAACACACATAAACAAACAAA
pADH1	CAGTTCGAGTTTATCATTATCAATACTGCCATTTCAAAGAATACGTAAATAATTAATAG<br>TAGTGATTTTCCTAACTTTATTTAGTCAAAAAATTAGCCTTTTAATTCTGCTGTAACCC<br>GTACATGCCCAAAATAGGGGGCGGGTTACACAGAATATATAACATCGTAGGTGTCTG<br>GGTGAACAGTTTATTCCTGGCATCCACTAAATATAATGGAGCCCGCTTTTTAAGCTG<br>GCATCCAGAAAAAAAAAGAATCCCAGCACCAAAATATTGTTTTCTTCACCAACCATC<br>AGTTCATAGGTCCATTCTCTTAGCGCAACTACAGAGAACAGGGGCACAAACAGGCA<br>AAAAACGGGCACAACCTCAATGGAGTGATGCAACCTGCCTGGAGTAAATGATGAC<br>ACAAGGCAATTGACCCACGCATGTATCTATCTCATTTTCTTACACCTTCTATTACCTTC<br>TGCTCTCTCTGATTTGGAAAAAGCTGAAAAAAAAGGTTGAAACCAGTTCCCTGAAA<br>TTATTCCCCTACTTGACTAATAAGTATATAAAGACGGTAGGTATTGATTGTAATTCTGT<br>AAATCTATTTCTTAAACTTCTTAAATTCTACTTTTATAGTTAGTCTTTTTTTTAGTTTTA<br>AAACACCAAGAACTTAGTTTCGAATAAACACACATAAACAAACAAA
pSNR52	CTTTGAAAAGATAATGTATGATTATGCTTTCACTCATATTTATACAGAAACTTGATGTT<br>TTCTTTCGAGTATATACAAGGTGATTACATGTACGTTTGAAGTACAACTCTAGATTTT<br>GTAGTGCCCTCTTGGGCTAGCGGTAAAGGTGCGCATTTTTTCACACCCTACAATGTT<br>CTGTTCAAAAGATTTTGGTCAAACGCTGTAGAAGTGAAAGTTGGTGCGCATGTTTC<br>GGCGTTCGAAACTTCTCCGCAGTGAAAGATAAATGATC

DEG1t_pCYC1noTATA	AATAATATATAAACCTGTATAATATAACCTTGAAGACTATATTTCTTTTCttcTTTCCTTATACATtAGGACCTTTGCAGCATAAATTACTATACTTCTATAGACACACAAACACAAATACACACACTAAATTAATA
Tsynth8.1_pCYC1noTATA	TTTCCCTATATAAACTCATTTACTTATGTAGGAATAAAGAGTATCATCTTTCAAATTCTTTCCTTATACATTAGGACCTTTGCAGCATAAATTACTATACTTCTATAGACACACAAACACAAATACACACACTAAATTAATA
yEBFP2	atgGTTTCCAAGGGTGAAGAATTATTCACCGGTGTTGTCCCTATTTTGGTCGAATTGGACGGTGATGTCAACGGTCACAAGTTCTCCGTTAGAGGTGAAGGTGAAGGTGACGCTACCAACGGTAAGTTGACCTTGAAGTTCATCTGTACCACTGGTAAGTTGCCTGTTCCTTGGCCAACTTTGGTTACGACTTTGTCTCACGGTGTTCAATGTTTCGCCCGTTACCCAGATCACATGAAGCAACATGATTTCTTTAAATCCGCCATGCCAGAAGGTTACGTTCAAGAAAGAACTATCTTCTTCAAGGACGATGGTACTTACAAAACTAGAGCCGAAGTCAAGTTCGAAGGTGATACTTTAGTCAATAGAATCGAATTGAAGGGTGTTGATTTCAAGGAAGATGGTAACATTTTGGGTCACAAGTTAGAATACAATTTCAACTCCCATAATATTTACATTATGGCTGTTAAGCAAAAGAACGGTATTAAGGTCAACTTTAAAATCAGACACAACGTTGAAGACGGTTCTGTCCAATTAGCTGACCACTACCAACAAAACACTCCAATTGGTGACGGTCCAGTCTTATTACCAGATTCTCATTACTTGTCTACCCAATCTGTCTTGTCTAAGGACCCAAATGAAAAGAGAGATCACATGGTCTTGTTAGAATTTCGTACTGCTGCTGGTATCACTTTGGGTATGGACGAATTGTACAAGtaa
yEGFPgg	atgTCTAAAGGTGAAGAATTATTCACTGGTGTTGTCCCAATTTTGGTTGAATTAGATGGTGATGTTAATGGTCACAAATTTTCTGTCTCCGGTGAAGGTGAAGGTGATGCTACTTACGGTAAATTGACCTTAAAATTTATTTGTACTACTGGTAAATTGCCAGTTCCATGGCCAACCTTAGTCACTACTTTCGGTTATGGTGTTCAATGTTTTGCGAGATACCCAGATCATATGAAACAACATGACTTTTTCAAGTCTGCCATGCCAGAAGGTTATGTTCAAGAAAGAACTATTTTTTTCAAAGATGACGGTAACTACAAGACCAGAGCTGAAGTCAAGTTTGAAGGTGATACCTTAGTTAATAGAATCGAATTAAAAGGTATTGATTTTAAAGAAGATGGTAACATTTTAGGTCACAAATTGGAATACAACTATAACTCTCACAATGTTTACATCATGGCTGACAAACAAAAGAATGGTATCAAAGTTAACTTCAAAATTAGACACAACATTGAAGATGGTTCTGTTCAATTAGCTGACCATTATCAACAAAATACTCCAATTGGTGATGGTCCAGTCTTGTTACCAGACAACCATTACTTATCCACTCAATCTGCCTTATCCAAAGATCCAAACGAAAAGAGgGACCACATGGTCTTGTTAGAATTTGTTACTGCTGCTGGTATTACCCATGGTATGGATGAATTGTACAAAtaa
yo_SpyCas9	atgGACAAGAAGTATTCTATCGGACTGGACATCGGGACTAATAGCGTCGGGTGGGCCGTCATCACTGACGAGTACAAGGTGCCCTCTAAGAAGTTCAAGGTGCTCGGGAACACCGACCGGCATTCCATCAAGAAAAATCTGATCGGAGCTCTCCTCTTTGATTCAGGGGAGACCGCTGAAGCAACCCGCCTCAAGCGGACTGCTAGACGGCGGTACACCAGGAGGAAGAACCGGATTTGTTACCTTCAAGAGATATTCTCCAACGAAATGGCAAAGGTCGACGACAGCTTCTTCCATAGGCTGGAAGAATCATTCCTCGTGGAAGAGGATAAGAAGCATGAACGGCATCCCATCTTCGGTAATATCGTCGACGAGGTGGCCTATCACGAGAAATACCCAACCATCTACCATCTTCGCAAAAAGCTGGTGGACTCAACCGACAAGGCAGACCTCCGGCTTATCTACCTGGCCCTGGCCCACATGATTAAGTTCAGAGGCCACTTCCTGATCGAGGGCGACCTCAATCCTGACAATAGCGATGTGGATAAACTGTTCATCCAGCTGGTGCAGACTTACAACCAGCTCTTTGAAGAGAACCCCATCAATGCAAGCGGAGTCGATGCCAAGGCCATTCTGTCAGCCCGGCTGTCAAAGAGCCGCAGACTTGAGAATCTTATCGCTCAGCTGCCGGGTGAAAAGAAAAATGGACTGTTCGGGAACCTGATTGCTCTTTCACTTGGGCTGACTCCCAATTTCAAGTCTAATTTCGACCTGGCAGAGGATGCCAAGCTGCAACTGTCCAAGGACACCTATGATGACGATCTCGACAACCTCCTGGCCCAG

	ATCGGTGACCAATACGCCGACCTTTTCCTTGCTGCTAAGAATCTTTCTGACGCCATCC<br>TGCTGTCTGACATTCTCCGCGTGAACACTGAAATCACCAAGGCCCCTCTTTCAGCTT<br>CAATGATTAAGCGGTATGATGAGCACCACCAGGACCTGACCCTGCTTAAGGCACTC<br>GTCCGGCAGCAGCTTCCGGAGAAGTACAAGGAAATCTTCTTTGACCAGTCAAAGA<br>ATGGATACGCCGGCTACATCGACGGAGGTGCCTCCCAAGAGGAATTTTATAAGTTTA<br>TCAAACCTATCCTTGAGAAGATGGACGGCACCGAAGAGCTCCTCGTGAAACTGAAT<br>CGGGAGGATCTGCTGCGGAAGCAGCGCACTTTCGACAATGGGAGCATTCCCCACC<br>AGATCCATCTTGGGGAGCTTCACGCCATCCTTCGGCGCCAAGAGGACTTCTACCCC<br>TTTCTTAAGGACAACAGGGAGAAGATTGAGAAAATTCTCACTTTCCGCATCCCCTAC<br>TACGTGGGACCCCTCGCCAGAGGAAATAGCCGGTTTGCTTGGATGACCAGAAAGT<br>CAGAAGAAACTATCACTCCCTGGAACTTCGAAGAGGTGGTGGACAAGGGAGCCAG<br>CGCTCAGTCATTCATCGAACGGATGACTAACTTCGATAAGAACCTCCCCAATGAGAA<br>GGTCCTGCCGAAACATTCCCTGCTCTACGAGTACTTTACCGTGTACAACGAGCTGAC<br>CAAGGTGAAATATGTCACCGAAGGGATGAGGAAGCCCGCATTCCTGTCAGGCGAA<br>CAAAAGAAGGCAATTGTGGACCTTCTGTTCAAGACCAATAGAAAGGTGACCGTGA<br>AGCAGCTGAAGGAGGACTATTTCAAGAAAATTGAATGCTTCGACTCTGTGGAGATT<br>AGCGGGGTCGAAGATCGGTTCAACGCAAGCCTGGGTACCTACCATGATCTGCTTAA<br>GATCATCAAGGACAAGGATTTTCTGGACAATGAGGAGAAAGAGGACATCCTTGAG<br>GACATTGTCCTGACTCTCACTCTGTTCGAGGACCGGGAAATGATCGAGGAGAGGCT<br>TAAGACCTACGCCCATCTGTTCGACGATAAAGTGATGAAGCAACTTAAACGGAGAA<br>GATATACCGGATGGGGACGCCTTAGCCGCAAACTCATCAACGGAATCCGGGACAAA<br>CAGAGCGGAAAGACCATTCTTGATTTCCTTAAGAGCGACGGATTCGCTAATCGCAA<br>CTTCATGCAACTTATCCATGATGATTCCCTGACCTTTAAGGAGGACATCCAGAAGGC<br>CCAAGTGTCTGGACAAGGTGACTCACTGCACGAGCATATCGCAAATCTGGCTGGTT<br>CACCCGCTATTAAGAAGGGTATTCTCCAGACCGTGAAAGTCGTGGACGAGCTGGTC<br>AAGGTGATGGGTCGCCATAAACCAGAGAACATTGTCATCGAGATGGCCAGGGAAA<br>ACCAGACTACCCAGAAGGGACAGAAGAACAGCAGGGAGCGGATGAAAAGAATTG<br>AGGAAGGGATTAAGGAGCTCGGGTCACAGATCCTTAAAGAGCACCCGGTGGAAA<br>ACACCCAGCTTCAGAATGAGAAGCTCTATCTGTACTACCTTCAAAATGGACGCGATA<br>TGTATGTGGACCAAGAGCTTGATATCAACAGGCTCTCAGACTACGACGTGGACCAC<br>ATCGTCCCTCAGAGCTTCCTCAAAGACGACTCAATTGACAATAAGGTGCTGACTCGC<br>TCAGACAAGAACCGGGGAAAGTCAGATAACGTGCCCTCAGAGGAAGTCGTGAAA<br>AAGATGAAGAACTATTGGCGCCAGCTTCTGAACGCAAAGCTAATCACTCAGCGGAA<br>GTTCGACAATCTCACTAAGGCTGAGAGGGGCGGACTGAGCGAACTGGACAAAGC<br>AGGATTCATTAAACGGCAACTTGTGGAGACTCGGCAGATTACTAAACATGTAGCCC<br>AAATCCTTGACTCACGCATGAATACCAAGTACGACGAAAACGACAAACTTATCCGCG<br>AGGTGAAGGTGATTACCCTGAAGTCCAAGCTGGTCAGCGATTTCAGAAAGGACTT<br>TCAATTCTACAAAGTGCGGGAGATCAATAACTATCATCATGCTCATGACGCATATCTG<br>AATGCCGTGGTGGGAACCGCCCTAATCAAGAAGTACCCAAAGCTGGAAAGCGAGT<br>TCGTGTACGGAGACTACAAGGTCTACGACGTGCGCAAGATGATTGCCAAATCTGAG<br>CAGGAGATCGGAAAGGCCACCGCAAAGTACTTCTTCTACAGCAACATCATGAATTT<br>CTTCAAGACCGAAATCACCCTTGCAAACGGTGAGATCCGGAAGAGGCCGCTCATC<br>GAGACTAATGGGGAGACTGGCGAAATCGTGTGGGACAAGGGCAGAGATTTCGCTA<br>CCGTGCGCAAAGTGCTTTCTATGCCTCAAGTGAACATCGTGAAGAAAACCGAGGTG<br>CAAACCGGAGGCTTTTCTAAGGAATCAATCCTCCCCAAGCGCAACTCCGACAAGCT<br>CATTGCAAGGAAGAAGGATTGGGACCCTAAGAAGTACGGCGGATTCGATTCACCA<br>ACTGTGGCTTATTCTGTCCTGGTCGTGGCTAAGGTGGAAAAAGGAAAGTCTAAGAA<br>GCTCAAGAGCGTGAAGGAACTGCTGGGTATCACCATTATGGAGCGCAGCTCCTTCG<br>AGAAGAACCCAATTGACTTTCTCGAAGCCAAAGGTTACAAGGAAGTCAAGAAGGA<br>CCTTATCATCAAGCTCCCAAAGTATAGCCTGTTCGAACTGGAGAATGGGCGGAAGC<br>GGATGCTCGCCTCCGCTGGCGAACTTCAGAAGGGTAATGAGCTGGCTCTCCCCTCC<br>AAGTACGTGAATTTCCTCTACCTTGCAAGCCATTACGAGAAGCTGAAGGGGAGCCC<br>CGAGGACAACGAGCAAAAGCAACTGTTTGTGGAGCAGCATAAGCATTATCTGGAC<br>GAGATCATTGAGCAGATTTCCGAGTTTTCTAAACGCGTCATTCTCGCTGATGCCAAC

	CTCGATAAAGTCCTTAGCGCATACAATAAGCACAGAGACAAACCAATTCGGGAGCA<br>GGCTGAGAATATCATCCACCTGTTCACCCTCACCAATCTTGGTGCCCCTGCCGCATTC<br>AAGTACTTCGACACCACCATCGACCGGAAACGCTATACCTCCACCAAAGAAGTGCT<br>GGACGCCACCCTCATCCACCAGAGCATCACCGGACTTTACGAAACTCGGATTGACC<br>TCTCACAGCTCGGAGGGGATGAGtaa
yo_dNme1Cas9	atgGCTGCTTTTAAACCAAATTCTATTAATTATATTTTGGGTTTGGCTATTGGTATTGCT<br>TCTGTTGGTTGGGCTATGGTTGAAATCGATGAGGAAGAAAATCCAATTAGATTGATT<br>GATTTGGGTGTCAGAGTTTTTGAAAGAGCTGAAGTTCCAAAGACTGGTGATTCTTT<br>GGCTATGGCTAGAAGATTGGCTAGATCTGTTAGAAGATTGACTAGAAGGAGGGCTC<br>ATAGATTGTTGAGAACTAGAAGATTATTGAAGAGAGAAGGTGTTTTGCAAGCTGCT<br>AATTTTGATGAAAATGGTTTGATTAAATCTTTGCCAAATACTCCATGGCAATTGAGA<br>GCTGCAGCTTTGGATAGAAAATTGACTCCTTTGGAATGGTCTGCTGTTTTGTTGCAT<br>TTGATTAAACATAGAGGTTATTTGTCTCAAAGAAAAAATGAAGGTGAAACTGCTGAT<br>AAAGAATTGGGTGCTTTGTTGAAGGGTGTTGCTGGTAATGCTCATGCTTTGCAAAC<br>TGGTGATTTTAGAACTCCTGCTGAATTGGCTTTGAATAAATTTGAAAAAGAATCTGG<br>TCATATTAGAAATCAAAGATCTGATTACTCACATACTTTTTCTAGGAAGGATTTGCAA<br>GCTGAATTGATTTTATTGTTTGAAAAACAAAAAGAATTTGGTAATCCACATGTTTCTG<br>GTGGTTTGAAAGAAGGTATTGAAACTTTGTTGATGACTCAAAGACCTGCTTTGTCT<br>GGTGATGCTGTTCAAAAAATGTTGGGTCATTGTACTTTTGAACCTGCTGAACCAAAA<br>GCTGCTAAAAACACATATACTGCTGAAAGGTTTATTTGGTTAACTAAGTTGAATAATT<br>TGAGAATTTTGGAACAAGGTTCTGAAAGACCATTGACTGATACTGAAAGAGCTACA<br>TTGATGGATGAACCTTATAGGAAATCTAAATTGACTTATGCTCAAGCTAGAAAATTAT<br>TAGGTTTGGAAGATACTGCTTTTTTTAAAGGTTTGAGATATGGTAAAGATAACGCAG<br>AAGCTTCTACTTTGATGGAAATGAAAGCTTATCATGCTATTTCAAGAGCTTTGGAAA<br>AAGAAGGTTTGAAAGATAAAAAATCTCCATTGAATTTGTCTCCTGAATTGCAAGATG<br>AAATTGGTACAGCTTTTTCTTTGTTTAAGACAGATGAAGATATTACTGGTAGATTGA<br>AAGATAGAATTCAACCTGAAATTTTAGAAGCATTGTTGAAACATATCTCATTTGATAA<br>ATTTGTTCAAATTTCTTTGAAAGCTTTGAGAAGAATTGTTCCATTGATGGAACAAGG<br>TAAAAGATATGATGAAGCATGTGCTGAAATTTATGGTGATCATTATGGTAAAAAAAA<br>CACTGAAGAAAAAATTTATTTGCCACCAATTCCTGCTGATGAAATTAGAAATCCTGT<br>TGTTTTGAGAGCTTTGTCACAAGCAAGAAAAGTTATCAATGGTGTTGTTAGAAGAT<br>ATGGTTCTCCTGCTAGAATTCATATTGAAACTGCAAGGGAGGTTGGTAAGTCTTTTA<br>AAGATAGAAAGGAAATTGAAAAAAGACAAGAAGAGAATAGAAAAGACAGAGAAA<br>AAGCTGCAGCTAAATTTAGGGAATACTTTCCTAACTTCGTCGGTGAACCAAAATCTA<br>AGGACATTTTGAAATTGAGATTGTATGAACAACAACACGGAAAATGTTTGTATTCTG<br>GTAAAGAGATCAATTTAGGAAGATTGAATGAAAAAGGTTATGTTGAAATTGATGCT<br>GCTTTGCCATTTTCAAGAACTTGGGATGACTCTTTTAATAATAAAGTTTTGGTTTTGG<br>GTTCTGAAAACCAAAATAAAGGTAATCAAACTCCTTATGAATATTTTAACGGTAAGG<br>ATAATTCAAGAGAATGGCAAGAATTTAAAGCTAGAGTTGAAACATCAAGATTTCCA<br>AGATCTAAGAAACAAAGAATTTTGTTGCAGAAGTTTGACGAGGATGGTTTTAAAGA<br>GAGAAACTTAAACGACACTAGATACGTTAATAGATTTTTGTGTCAATTTGTTGCTGAT<br>AGAATGAGATTGACTGGTAAAGGTAAAAAAAGAGTTTTTGCTTCTAATGGTCAAAT<br>TACTAATTTGTTGAGAGGTTTTTGGGGTTTGAGAAAAGTTAGAGCTGAAAATGATA<br>GACATCATGCTTTGGATGCTGTTGTCGTTGCTTGTTCAACTGTTGCTATGCAGCAAA<br>AAATTACTAGGTTTGTTAGATATAAAGAGATGAATGCTTTTGATGGTAAAACTATTGA<br>TAAAGAAACTGGTGAAGTTTTGCATCAAAAAACTCATTTTCCTCAACCATGGGAATT<br>TTTTGCTCAAGAAGTTATGATTAGAGTTTTTGGAAAACCTGATGGTAAGCCTGAATT<br>TGAAGAAGCTGATACTTTGGAAAAATTGAGAACTTTGTTGGCTGAAAAATTGTCTT<br>CAAGACCTGAAGCTGTTCATGAATATGTTACTCCATTGTTTGTTTCAAGGGCTCCAA<br>ATAGAAAAATGTCTGGTCAAGGTCACATGGAAACTGTTAAATCTGCTAAAAGATTG<br>GATGAAGGTGTTTCTGTTTTGAGGGTTCCATTGACACAATTGAAATTGAAAGATTTG<br>GAAAAAATGGTCAATAGAGAAAGAGAACCAAAATTGTATGAAGCTTTGAAAGCTA

	GATTGGAAGCTCATAAAGATGATCCTGCTAAAGCTTTTGCTGAACCATTTTATAAATA<br>TGATAAAGCTGGTAATAGAACTCAACAAGTTAAAGCTGTTAGAGTTGAACAAGTTC<br>AAAAAACTGGTGTTTGGGTTAGAAATCATAATGGTATTGCTGATAATGCTACTATGGT<br>TAGAGTTGATGTCTTTGAAAAAGGAGATAAATATTACTTGGTTCCAATTTATTCTTGG<br>CAAGTTGCTAAAGGTATTTTGCCTGATAGAGCTGTTGTTCAAGGTAAAGACGAAGA<br>AGACTGGCAGTTGATTGATGATTCTTTTAACTTTAAATTTTCTTTGCATCCTAACGAT<br>TTGGTTGAAGTCATTACTAAAAAAGCTAGAATGTTTGGTTATTTTGCATCTTGTCACA<br>GAGGTACTGGTAACATTAATATCAGAATTCATGATTTGGATCATAAAATCGGTAAGAA<br>TGGTATTTTGGAAGGTATTGGTGTTAAAACTGCTTTGTCTTTTCAAAAATATCAAATT<br>GACGAATTGGGAAAAGAAATTAGGCCATGTAGATTGAAAAAAAGACCACCTGTTA<br>GAtaa
yo_d$_{16A}$Nme1Cas9<br>(The marked ***cat*** is the A588H mutation with respect to yo_dNme1Cas9)	atgGCTGCTTTTAAACCAAATTCTATTAATTATATTTTGGGTTTGGCTATTGGTATTGCT<br>TCTGTTGGTTGGGCTATGGTTGAAATCGATGAGGAAGAAAATCCAATTAGATTGATT<br>GATTTGGGTGTCAGAGTTTTTGAAAGAGCTGAAGTTCCAAAGACTGGTGATTCTTT<br>GGCTATGGCTAGAAGATTGGCTAGATCTGTTAGAAGATTGACTAGAAGGAGGGCTC<br>ATAGATTGTTGAGAACTAGAAGATTATTGAAGAGAGAAGGTGTTTTGCAAGCTGCT<br>AATTTTGATGAAAATGGTTTGATTAAATCTTTGCCAAATACTCCATGGCAATTGAGA<br>GCTGCAGCTTTGGATAGAAAATTGACTCCTTTGGAATGGTCTGCTGTTTTGTTGCAT<br>TTGATTAAACATAGAGGTTATTTGTCTCAAAGAAAAAATGAAGGTGAAACTGCTGAT<br>AAAGAATTGGGTGCTTTGTTGAAGGGTGTTGCTGGTAATGCTCATGCTTTGCAAAC<br>TGGTGATTTTAGAACTCCTGCTGAATTGGCTTTGAATAAATTTGAAAAAGAATCTGG<br>TCATATTAGAAATCAAAGATCTGATTACTCACATACTTTTTCTAGGAAGGATTTGCAA<br>GCTGAATTGATTTTATTGTTTGAAAAACAAAAAGAATTTGGTAATCCACATGTTTCTG<br>GTGGTTTGAAAGAAGGTATTGAAACTTTGTTGATGACTCAAAGACCTGCTTTGTCT<br>GGTGATGCTGTTCAAAAAATGTTGGGTCATTGTACTTTTGAACCTGCTGAACCAAAA<br>GCTGCTAAAAACACATATACTGCTGAAAGGTTTATTTGGTTAACTAAGTTGAATAATT<br>TGAGAATTTTGGAACAAGGTTCTGAAAGACCATTGACTGATACTGAAAGAGCTACA<br>TTGATGGATGAACCTTATAGGAAATCTAAATTGACTTATGCTCAAGCTAGAAAATTAT<br>TAGGTTTGGAAGATACTGCTTTTTTTAAAGGTTTGAGATATGGTAAAGATAACGCAG<br>AAGCTTCTACTTTGATGGAAATGAAAGCTTATCATGCTATTTCAAGAGCTTTGGAAA<br>AAGAAGGTTTGAAAGATAAAAAATCTCCATTGAATTTGTCTCCTGAATTGCAAGATG<br>AAATTGGTACAGCTTTTTCTTTGTTTAAGACAGATGAAGATATTACTGGTAGATTGA<br>AAGATAGAATTCAACCTGAAATTTTAGAAGCATTGTTGAAACATATCTCATTTGATAA<br>ATTTGTTCAAATTTCTTTGAAAGCTTTGAGAAGAATTGTTCCATTGATGGAACAAGG<br>TAAAAGATATGATGAAGCATGTGCTGAAATTTATGGTGATCATTATGGTAAAAAAAA<br>CACTGAAGAAAAAATTTATTTGCCACCAATTCCTGCTGATGAAATTAGAAATCCTGT<br>TGTTTTGAGAGCTTTGTCACAAGCAAGAAAAGTTATCAATGGTGTTGTTAGAAGAT<br>ATGGTTCTCCTGCTAGAATTCATATTGAAACTGCAAGGGAGGTTGGTAAGTCTTTTA<br>AAGATAGAAAGGAAATTGAAAAAAGACAAGAAGAGAATAGAAAAGACAGAGAAA<br>AAGCTGCAGCTAAATTTAGGGAATACTTTCCTAACTTCGTCGGTGAACCAAAATCTA<br>AGGACATTTTGAAATTGAGATTGTATGAACAACAACACGGAAAATGTTTGTATTCTG<br>GTAAAGAGATCAATTTAGGAAGATTGAATGAAAAAGGTTATGTTGAAATTGAT***cat***G<br>CTTTGCCATTTTCAAGAACTTGGGATGACTCTTTTAATAATAAAGTTTTGGTTTTGGG<br>TTCTGAAAACCAAAATAAAGGTAATCAAACTCCTTATGAATATTTTAACGGTAAGGAT<br>AATTCAAGAGAATGGCAAGAATTTAAAGCTAGAGTTGAAACATCAAGATTTCCAAG<br>ATCTAAGAAACAAAGAATTTTGTTGCAGAAGTTTGACGAGGATGGTTTTAAAGAGA<br>GAAACTTAAACGACACTAGATACGTTAATAGATTTTTGTGTCAATTTGTTGCTGATAG<br>AATGAGATTGACTGGTAAAGGTAAAAAAAGAGTTTTTGCTTCTAATGGTCAAATTAC<br>TAATTTGTTGAGAGGTTTTTGGGGTTTGAGAAAAGTTAGAGCTGAAAATGATAGAC<br>ATCATGCTTTGGATGCTGTTGTCGTTGCTTGTTCAACTGTTGCTATGCAGCAAAAAAT<br>TACTAGGTTTGTTAGATATAAAGAGATGAATGCTTTTGATGGTAAAACTATTGATAAA<br>GAAACTGGTGAAGTTTTGCATCAAAAAACTCATTTTCCTCAACCATGGGAATTTTTT

	GCTCAAGAAGTTATGATTAGAGTTTTTGGAAAACCTGATGGTAAGCCTGAATTTGA<br>AGAAGCTGATACTTTGGAAAAAATTGAGAACTTTGTTGGCTGAAAAATTGTCTTCAA<br>GACCTGAAGCTGTTCATGAATATGTTACTCCATTGTTTGTTTCAAGGGCTCCAAATAG<br>AAAAATGTCTGGTCAAGGTCACATGGAAACTGTTAAATCTGCTAAAAGATTGGATG<br>AAGGTGTTTCTGTTTTGAGGGTTCCATTGACACAATTGAAATTGAAAGATTTGGAA<br>AAAATGGTCAATAGAGAAAGAGAACCAAAATTGTATGAAGCTTTGAAAGCTAGATT<br>GGAAGCTCATAAAGATGATCCTGCTAAAGCTTTTGCTGAACCATTTTATAAATATGAT<br>AAAGCTGGTAATAGAACTCAACAAGTTAAAGCTGTTAGAGTTGAACAAGTTCAAAA<br>AACTGGTGTTTGGGTTAGAAATCATAATGGTATTGCTGATAATGCTACTATGGTTAGA<br>GTTGATGTCTTTGAAAAAGGAGATAAATATTACTTGGTTCCAATTTATTCTTGGCAAG<br>TTGCTAAAGGTATTTTGCCTGATAGAGCTGTTGTTCAAGGTAAAGACGAAGAAGAC<br>TGGCAGTTGATTGATGATTCTTTTAACTTTAAATTTTCTTTGCATCCTAACGATTTGGT<br>TGAAGTCATTACTAAAAAAGCTAGAATGTTTGGTTATTTTGCATCTTGTCACAGAGG<br>TACTGGTAACATTAATATCAGAATTCATGATTTGGATCATAAAATCGGTAAGAATGGT<br>ATTTTGGAAGGTATTGGTGTTAAAACTGCTTTGTCTTTTCAAAAATATCAAATTGACG<br>AATTGGGAAAAGAAATTAGGCCATGTAGATTGAAAAAAAGACCACCTGTTAGAtaa
yo_Nme1Cas9<br>(The marked ***gat*** and ***cat*** are the A16D and A588D mutations with respect to yo_dNme1Cas9)	atgGCTGCTTTTAAACCAAATTCTATTAATTATATTTTGGGTTTG***gat***ATTGGTATTGCT<br>TCTGTTGGTTGGGCTATGGTTGAAATCGATGAGGAAGAAAATCCAATTAGATTGATT<br>GATTTGGGTGTCAGAGTTTTTGAAAGAGCTGAAGTTCCAAAGACTGGTGATTCTTT<br>GGCTATGGCTAGAAGATTGGCTAGATCTGTTAGAAGATTGACTAGAAGGAGGGCTC<br>ATAGATTGTTGAGAACTAGAAGATTATTGAAGAGAGAAGGTGTTTTGCAAGCTGCT<br>AATTTTGATGAAAATGGTTTGATTAAATCTTTGCCAAATACTCCATGGCAATTGAGA<br>GCTGCAGCTTTGGATAGAAAATTGACTCCTTTGGAATGGTCTGCTGTTTTGTTGCAT<br>TTGATTAAACATAGAGGTTATTTGTCTCAAAGAAAAAATGAAGGTGAAACTGCTGAT<br>AAAGAATTGGGTGCTTTGTTGAAGGGTGTTGCTGGTAATGCTCATGCTTTGCAAAC<br>TGGTGATTTTAGAACTCCTGCTGAATTGGCTTTGAATAAATTTGAAAAAGAATCTGG<br>TCATATTAGAAATCAAAGATCTGATTACTCACATACTTTTTCTAGGAAGGATTTGCAA<br>GCTGAATTGATTTTATTGTTTGAAAAACAAAAAGAATTTGGTAATCCACATGTTTCTG<br>GTGGTTTGAAAGAAGGTATTGAAACTTTGTTGATGACTCAAAGACCTGCTTTGTCT<br>GGTGATGCTGTTCAAAAAATGTTGGGTCATTGTACTTTTGAACCTGCTGAACCAAAA<br>GCTGCTAAAAACACATATACTGCTGAAAGGTTTATTTGGTTAACTAAGTTGAATAATT<br>TGAGAATTTTGGAACAAGGTTCTGAAAGACCATTGACTGATACTGAAAGAGCTACA<br>TTGATGGATGAACCTTATAGGAAATCTAAATTGACTTATGCTCAAGCTAGAAAATTAT<br>TAGGTTTGGAAGATACTGCTTTTTTTAAAGGTTTGAGATATGGTAAAGATAACGCAG<br>AAGCTTCTACTTTGATGGAAATGAAAGCTTATCATGCTATTTCAAGAGCTTTGGAAA<br>AAGAAGGTTTGAAAGATAAAAAATCTCCATTGAATTTGTCTCCTGAATTGCAAGATG<br>AAATTGGTACAGCTTTTTCTTTGTTTAAGACAGATGAAGATATTACTGGTAGATTGA<br>AAGATAGAATTCAACCTGAAATTTTAGAAGCATTGTTGAAACATATCTCATTTGATAA<br>ATTTGTTCAAATTTCTTTGAAAGCTTTGAGAAGAATTGTTCCATTGATGGAACAAGG<br>TAAAAGATATGATGAAGCATGTGCTGAAATTTATGGTGATCATTATGGTAAAAAAA<br>CACTGAAGAAAAAATTTATTTGCCACCAATTCCTGCTGATGAAATTAGAAATCCTGT<br>TGTTTTGAGAGCTTTGTCACAAGCAAGAAAAGTTATCAATGGTGTTGTTAGAAGAT<br>ATGGTTCTCCTGCTAGAATTCATATTGAAACTGCAAGGGAGGTTGGTAAGTCTTTTA<br>AAGATAGAAAGGAAATTGAAAAAAGACAAGAAGAGAATAGAAAAGACAGAGAAA<br>AAGCTGCAGCTAAATTTAGGGAATACTTTCCTAACTTCGTCGGTGAACCAAAATCTA<br>AGGACATTTTGAAATTGAGATTGTATGAACAACAACACGGAAAATGTTTGTATTCTG<br>GTAAAGAGATCAATTTAGGAAGATTGAATGAAAAAGGTTATGTTGAAATTGAT***cat***G<br>CTTTGCCATTTTCAAGAACTTGGGATGACTCTTTTAATAATAAAGTTTTGGTTTTGGG<br>TTCTGAAAACCAAAATAAAGGTAATCAAACTCCTTATGAATATTTTAACGGTAAGGAT<br>AATTCAAGAGAATGGCAAGAATTTAAAGCTAGAGTTGAAACATCAAGATTTCCAAG<br>ATCTAAGAAACAAAGAATTTTGTTGCAGAAGTTTGACGAGGATGGTTTTAAAGAGA<br>GAAACTTAAACGACACTAGATACGTTAATAGATTTTTGTGTCAATTTGTTGCTGATAG

	AATGAGATTGACTGGTAAAGGTAAAAAAAGAGTTTTTGCTTCTAATGGTCAAATTAC<br>TAATTTGTTGAGAGGTTTTTGGGGTTTGAGAAAAGTTAGAGCTGAAAATGATAGAC<br>ATCATGCTTTGGATGCTGTTGTCGTTGCTTGTTCAACTGTTGCTATGCAGCAAAAAAT<br>TACTAGGTTTGTTAGATATAAAGAGATGAATGCTTTTGATGGTAAAACTATTGATAAA<br>GAAACTGGTGAAGTTTTGCATCAAAAAACTCATTTTCCTCAACCATGGGAATTTTTT<br>GCTCAAGAAGTTATGATTAGAGTTTTTGGAAAACCTGATGGTAAGCCTGAATTTGA<br>AGAAGCTGATACTTTGGAAAAATTGAGAACTTTGTTGGCTGAAAAATTGTCTTCAA<br>GACCTGAAGCTGTTCATGAATATGTTACTCCATTGTTTGTTTCAAGGGCTCCAAATAG<br>AAAAATGTCTGGTCAAGGTCACATGGAAACTGTTAAATCTGCTAAAAGATTGGATG<br>AAGGTGTTTCTGTTTTGAGGGTTCCATTGACACAATTGAAATTGAAAGATTTGGAA<br>AAAATGGTCAATAGAGAAAGAGAACCAAAATTGTATGAAGCTTTGAAAGCTAGATT<br>GGAAGCTCATAAAGATGATCCTGCTAAAGCTTTTGCTGAACCATTTTATAAATATGAT<br>AAAGCTGGTAATAGAACTCAACAAGTTAAAGCTGTTAGAGTTGAACAAGTTCAAAA<br>AACTGGTGTTTGGGTTAGAAATCATAATGGTATTGCTGATAATGCTACTATGGTTAGA<br>GTTGATGTCTTTGAAAAAGGAGATAAATATTACTTGGTTCCAATTTATTCTTGGCAAG<br>TTGCTAAAGGTATTTTGCCTGATAGAGCTGTTGTTCAAGGTAAAGACGAAGAAGAC<br>TGGCAGTTGATTGATGATTCTTTTAACTTTAAATTTTCTTTGCATCCTAACGATTTGGT<br>TGAAGTCATTACTAAAAAAGCTAGAATGTTTGGTTATTTTGCATCTTGTCACAGAGG<br>TACTGGTAACATTAATATCAGAATTCATGATTTGGATCATAAAATCGGTAAGAATGGT<br>ATTTTGGAAGGTATTGGTGTTAAAACTGCTTTGTCTTTTCAAAAATATCAAATTGACG<br>AATTGGGAAAAGAAATTAGGCCATGTAGATTGAAAAAAAGACCACCTGTTAGAtaa
yo_LwaCas13a	atgAAAGTTACAAAGGTTGACGGAATTTCACACAAAAAATATATTGAAGAGGGTA<br>AATTGGTTAAGTCAACTTCTGAGGAAAACAGAACTTCAGAAAGATTGTCTGAATT<br>GTTGTCTATAAGGTTAGATATATACATTAAGAATCCAGACAACGCTTCAGAGGAA<br>GAAAACAGAATTAGGAGAGAGAATTTGAAAAAATTTTTCTCTAATAAAGTTTTAC<br>ATTTGAAAGACTCTGTCTTGTACTTGAAGAACAGAAAGGAGAAAAATGCAGTCCA<br>AGATAAGAACTATTCTGAAGAGGATATTTCTGAGTACGATTTGAAAAATAAAAAC<br>TCTTTTTCAGTTTTGAAGAAAATTTTGTTGAATGAAGACGTTAATTCAGAGGAATT<br>GGAAATTTTTAGAAAAGATGTTGAAGCAAAATTGAACAAAATTAATTCTTTAAAAT<br>ATTCTTTTGAAGAAAATAAAGCTAACTACCAAAAAATTAACGAAAACAATGTTGA<br>AAAAGTTGGTGGAAAATCAAAGAGGAATATTATTTACGATTATTACAGAGAATCT<br>GCTAAGAGGAATGATTATATTAATAATGTTCAGGAGGCTTTCGACAAATTGTACA<br>AGAAAGAAGACATTGAGAAATTATTCTTTTTAATTGAGAATTCTAAAAAGCACGA<br>GAAATACAAAATTAGGGAATACTACCATAAGATTATTGGTAGGAAAAATGACAAA<br>GAAAATTTCGCTAAGATTATTTATGAAGAAATTCAAAATGTTAATAATATTAAAGA<br>ATTGATTGAAAAAATTCCAGATATGTCAGAATTGAAAAAGTCACAAGTTTTCTACA<br>AATACTACTTGGATAAAGAAGAATTGAATGACAAAAATATTAAGTACGCATTTTG<br>TCACTTCGTTGAAATTGAGATGTCTCAATTGTTGAAAAACTATGTTTATAAAAGGT<br>TATCAAATATATCTAATGATAAGATTAAAAGAATTTTTGAGTACCAAAATTTGAAG<br>AAATTAATTGAAAACAAATTGTTGAATAAATTGGATACTTATGTCAGAAATTGCG<br>GTAAGTATAATTACTACTTGCAGGTTGGTGAAATTGCTACATCTGATTTTATTGCT<br>AGAAATAGACAAAACGAAGCTTTCTTGAGAAATATAATTGGTGTTTCATCTGTTGC<br>TTATTTCTCATTAAGAAATATTTTAGAAACTGAGAACGAGAACGACATTACTGGAA<br>GGATGAGGGGTAAAACTGTTAAAAACAACAAAGGTGAAGAAAAGTATGTTTCAG<br>GAGAGGTTGACAAGATTTACAATGAAAATAAGCAAAATGAAGTTAAAGAGAATT<br>TAAAAATGTTCTACTCTTACGATTTCAACATGGACAACAAGAATGAAATTGAAGAT<br>TTTTTCGCAAACATTGACGAAGCTATTTCTTCTATTAGACACGGTATAGTTCACTTT<br>AACTTAGAATTGGAAGGTAAAGACATATTCGCATTTAAAAACATAGCTCCTTCAG<br>AGATTTCTAAGAAAATGTTTCAGAACGAAATAAACGAAAAGAAATTAAAATTAAA<br>AATATTTAAGCAATTAAATTCTGCTAATGTCTTTAATTACTACGAAAAAGATGTCAT<br>TATTAAATATTTGAAAAACACAAAATTTAATTTTGTTAATAAAAACATACCATTTGT<br>TCCATCTTTTACTAAATTATATAACAAAATAGAAGATTTAAGAAATACTTTGAAGTT

	CTTTTGGTCTGTTCCAAAAGATAAGGAAGAGAAGGATGCACAAATATATTTATTG<br>AAGAATATATATTATGGAGAGTTTTTAAATAAATTCGTCAAGAATTCAAAGGTCTT<br>TTTCAAAATTACTAATGAAGTTATTAAGATTAATAAGCAGAGAAATCAAAAAACT<br>GGTCATTATAAGTACCAGAAGTTTGAAAATATTGAAAAAACTGTTCCTGTCGAGT<br>ACTTAGCTATAATTCAATCAAGGGAAATGATTAACAATCAGGATAAAGAGGAGAA<br>AAATACATACATTGATTTTATACAACAAATCTTTTTAAAAGGATTCATTGACTACTT<br>GAATAAGAACAACTTGAAGTATATAGAGTCTAATAACAATAACGACAACAACGAT<br>ATTTTCTCAAAAATTAAAATTAAAAAAGATAACAAAGAAAAATACGACAAAATTTT<br>AAAAAATTACGAGAAGCATAACAGAAATAAGGAGATTCCACATGAAATTAACGA<br>ATTCGTCAGAGAAATTAAGTTAGGAAAAATATTGAAGTACACAGAAAACTTGAAC<br>ATGTTTTATTTGATTTTGAAATTGTTGAACCACAAAGAATTAACAAATTTAAAGGG<br>TTCTTTAGAAAAATATCAGTCTGCTAACAAAGAAGAGACATTCTCAGATGAATTG<br>GAATTGATTAATTTATTGAATTTGGACAACAATAGGGTTACAGAAGATTTCGAATT<br>GGAGGCTAATGAAATTGGTAAATTTTTGGATTTTAATGAGAATAAGATTAAGGAT<br>AGAAAGGAATTAAAAAAGTTCGATACAAATAAGATATACTTCGACGGTGAAAATA<br>TAATTAAACATAGAGCTTTTTACAACATTAAAAAGTACGGTATGTTGAATTTGTTG<br>GAGAAAATTGCTGACAAAGCAAAATATAAGATTTCTTTAAAAGAGTTAAAGGAAT<br>ACTCAAATAAGAAAAATGAGATTGAGAAAAATTATACTATGCAACAAAACTTGCA<br>TAGGAAATACGCTAGACCTAAAAAGGATGAGAAATTTAACGACGAGGATTATAA<br>AGAATACGAAAAGGCTATTGGTAACATTCAAAAATACACTCACTTGAAAAATAAG<br>GTTGAGTTTAACGAATTAAATTTATTACAAGGTTTGTTGTTAAAAATTTTACATAG<br>ATTGGTTGGTTACACTTCTATTTGGGAAAGAGATTTAAGGTTTAGATTAAAGGGT<br>GAATTTCCAGAGAACCATTACATTGAAGAGATTTTTAACTTCGATAATTCAAAAAA<br>CGTTAAATATAAATCAGGACAAATAGTTGAAAAGTACATTAATTTCTATAAAGAAT<br>TGTATAAGGATAACGTTGAGAAGAGATCTATTTACTCTGATAAAAAGGTCAAAAA<br>GTTAAAACAAGAAAAGAAAGATTTGTATATTAGAAACTACATTGCACATTTCAATT<br>ATATACCTCATGCTGAGATTTCTTTATTGGAAGTTTTAGAGAACTTGAGAAAATTG<br>TTGTCTTATGATAGAAAGTTAAAAAACGCTATAATGAAATCTATAGTTGACATTTT<br>AAAAGAGTATGGATTCGTTGCTACTTTTAAAATAGGTGCTGATAAGAAAATTGAA<br>ATTCAAACATTGGAATCTGAAAAAATTGTTCACTTAAAAAACTTGAAAAAGAAAA<br>AGTTAATGACTGACAGAAACTCAGAAGAGTTGTGCGAATTGGTTAAAGTTATGTT<br>CGAATATAAAGCTCTCGAGtaa
yo_RfxCas13d	atgGAAGCCAGTATCGAAAAGAAAAAAAGTTTCGCTAAAGGTATGGGTGTCAAGA<br>GTACCCTTGTCTCTGGATCTAAAGTTTACATGACCACTTTCGCCGAGGGATCTGAC<br>GCTAGGCTTGAAAAGATCGTCGAGGGAGACAGTATTAGAAGTGTTAATGAAGGT<br>GAAGCTTTTTCTGCCGAGATGGCCGACAAGAATGCCGGTTACAAAATCGGTAATG<br>CTAAGTTTTCTCACCCAAAAGGATACGCCGTCGTCGCCAACAACCCTCTTTACACT<br>GGACCAGTTCAGCAAGATATGTTGGGTCTTAAGGAAACCTTGGAGAAGAGATAC<br>TTCGGTGAATCTGCCGACGGTAACGACAACATCTGTATCCAAGTCATTCACAATAT<br>CTTGGACATCGAAAAGATCCTTGCCGAATACATCACCAATGCTGCCTACGCCGTCA<br>ATAACATCAGTGGATTGGACAAAGATATTATTGGTTTTGGTAAATTTAGTACTGTC<br>TACACCTATGACGAGTTTAAAGATCCAGAGCACCATAGAGCCGCCTTTAACAACA<br>ATGATAAGTTGATTAACGCCATCAAGGCCCAGTACGACGAATTTGACAACTTCCTT<br>GATAACCCTAGGTTGGGTTACTTCGGACAAGCCTTCTTCAGTAAAGAAGGAAGAA<br>ACTACATCATTAATTACGGAAACGAATGCTACGACATTCTTGCCCTTTTGTCTGGA<br>TTGAGGCATTGGGTCGTTCACAATAACGAGGAGGAGAGTAGGATCAGTAGGACT<br>TGGCTTTATAACTTGGATAAGAATTTGGATAATGAATATATCAGTACTTTGAATTA<br>CTTGTACGATAGAATCACCAATGAGTTGACCAATTCTTTTTCTAAGAATAGTGCTG<br>CCAACGTCAATTACATCGCTGAAACCTTGGGTATCAACCCAGCTGAGTTCGCTGA<br>GCAGTACTTTAGATTTTCTATTATGAAAGAACAAAAAAATTTGGGTTTTAATATCA<br>CTAAGTTGAGAGAGGTTATGTTGGATAGAAAGGACATGAGTGAAATTAGAAAAA<br>ACCACAAAGTTTTCGACAGTATCAGAACCAAAGTCTACACCATGATGGACTTTGTC

	ATTTACAGATATTACATTGAGGAAGATGCCAAGGTTGCCGCTGCCAACAAATCTTT<br>GCCAGACAACGAAAAGAGTTTGTCTGAGAAGGACATTTTCGTTATCAACTTGAGA<br>GGTTCTTTCAATGACGACCAGAAAGACGCCTTGTATTACGACGAGGCCAATAGAA<br>TCTGGAGGAAATTGGAAAATATCATGCACAATATCAAAGAATTTAGGGGAAATAA<br>GACTAGAGAGTACAAGAAAAAGGACGCCCCTAGGCTTCCTAGGATCTTGCCAGCT<br>GGAAGGGACGTTTCTGCCTTCAGTAAATTGATGTACGCCTTGACTATGTTTTTGGA<br>TGGTAAGGAAATCAATGACCTTTTGACTACTCTTATCAACAAATTTGACAATATCC<br>AAAGTTTCTTGAAGGTCATGCCTCTTATCGGTGTCAACGCTAAGTTCGTCGAGGA<br>GTACGCCTTCTTTAAAGATTCTGCTAAAATCGCTGATGAGCTTAGACTTATCAAAT<br>CTTTTGCCAGAATGGGAGAGCCAATCGCTGATGCTAGGAGGGCTATGTATATCGA<br>CGCCATTAGGATTCTTGGTACTAACCTTAGTTATGACGAGCTTAAAGCTTTGGCCG<br>ACACCTTCTCTCTTGATGAAAACGGTAATAAACTTAAAAAGGGAAAACATGGAAT<br>GAGAAATTTTATCATCAATAATGTCATCTCTAACAAAAGGTTCCACTACTTGATTA<br>GGTACGGAGATCCAGCCCATCTTCACGAGATCGCCAAGAACGAGGCCGTTGTCAA<br>GTTCGTCTTGGGAAGGATTGCCGACATCCAGAAGAAGCAAGGTCAGAACGGAAA<br>GAACCAGATCGACAGATACTACGAAACTTGTATTGGTAAAGATAAGGGTAAATCT<br>GTCAGTGAAAAGGTCGATGCCTTGACCAAGATCATCACCGGTATGAACTACGATC<br>AATTTGACAAGAAGAGAAGTGTTATTGAAGATACCGGAAGGGAGAACGCCGAAA<br>GAGAAAAGTTCAAAAAGATCATCTCTCTTTACCTTACTGTTATCTATCACATTTTGA<br>AAAACATCGTCAATATTAATGCCAGATATGTCATCGGATTCCACTGCGTCGAGAG<br>AGATGCCCAGTTGTATAAAGAAAAAGGTTACGACATTAATCTTAAAAAGTTGGAA<br>GAAAAGGGATTTAGTAGTGTTACCAAGCTTTGTGCTGGAATCGATGAAACCGCTC<br>CAGACAAAAGGAAGGACGTTGAAAAAGAAATGGCCGAGAGAGCCAAGGAGTCT<br>ATCGACAGTTTGGAGTCTGCCAACCCAAAGTTGTACGCTAATTATATCAAGTACAG<br>TGATGAAAAAAAAGCCGAGGAGTTCACTAGACAAATCAATAGAGAAAAAGCCAA<br>AACCGCCTTGAATGCTTACTTGAGGAATACCAAGTGGAACGTTATTATCAGAGAA<br>GATCTTTTGAGAATCGATAATAAGACTTGTACCCTTTTTAGAAACAAAGCTGTCCA<br>CTTGGAGGTCGCTAGATACGTCCACGCCTACATCAACGACATTGCTGAGGTCAAT<br>TCTTACTTTCAGTTGTACCATTACATTATGCAGAGGATCATTATGAATGAGAGATA<br>CGAGAAAAGTTCTGGTAAAGTTTCTGAATACTTCGACGCCGTCAACGATGAAAAA<br>AAGTACAACGATAGACTTCTTAAATTGTTGTGTGTCCCATTTGGTTACTGTATTCCA<br>AGATTTAAGAATTTGTCTATCGAAGCCCTTTTTGATAGAAACGAGGCTGCTAAGTT<br>CGACAAGGAAAAAAAGAAAGTTAGTGGAAATAGTGGTTCTGGTtaa
yo_RspWYL1<br>(with HAtag)	atgAAGATCGAGGAAGGAAAGGGTTATCCTTATGATGTTCCTGATTATGCAATGCTTA<br>TTCCTCCTTCAACATTTCTTCCTAAGCGCGACAAGAATGTACCTTATATTGCAGAAGT<br>TCAGTCGATACCGCTGTCACCCTCAGCATATTCCGTAATCATAAAGGACAAGTCCATC<br>TTTGAAACTTCACTTTCACCTAATGGCTCTGTCTCCATGTCTTCTTTCTTGACCTCCAT<br>CTTCGACAGCGCCTACATCGCCTCCTTAAAGTACAAGTCTGACGACAATTACAAGTA<br>CATAGGAATTCCTCTTCTTAACGCATTTGTTAAATGGCAGATAGAGGAGATCGACGA<br>CGGCTTAGACGACAAGTCCAAAGAAATAATAAAGTCGTACTTAATCAGTAAACTATC<br>AGCGAAATATGAGAAAACGAAAACAGAGAATGCAGTGAGGGTGCGCCTGTCTATA<br>TGCAGGGACCTCTACGACACTTTATCTAGTGACGACCTCTACTACGAGAATAAGGTA<br>TACTCGTCCACGTTACGGCGGTTCCTAAAGGCGGTTTATGAAGATTATGCACTCCTG<br>TCTGATTGTGAAAGGGAGCGGCTGATATTCGCAGACAATATAATAAAGATAAATGAG<br>GTCATCAAGCAGAATGGAAGTCGTTACTACTCCTTCATATACGCTTACTCCAATATGT<br>ACTCTCGCGAGAAGCGACGTATACGATTGATACCCTACCGCATCGTCAGCGACGAGT<br>ACAAGATGTACAATTATCTTGTTTGTCTTTCAGATGAGAAGTCTGCGGGAAAGGAG<br>TTCAAAGCAGATTCATGTAGAATTTCAAGACTATCTGGGTTGAGCATCGCTGAGAA<br>GTTATCCCAGAAGGAGTACAGCTCGGTCACGGAGTACGAGCGATTGAAGGAGGTA<br>CACGTGAAGTCTGTTAAACATTTATTGTCAGATCCTCGCTTCGGTAGCGACGAGTCT<br>GACATAAGTAAAGTGTATCTCACTGAGAAGGGCGTGGAGATGTTCGGTAAGATACT<br>ATACCAGAGACCTATTCTTAAAGGAAACGAGAAGCCAAAGCCTAACGCGGTCAATG

	AGTTCATATCTCCTCCTATTCAGGTGAAGTACTACTTTAACAAATTTGGAAAGGACG GTGTTATCCTGAGCCCAAGCGACAGCTTTGAAGAAATGAGAACACTTTATGTTGAA GGAGCAGAAGCATATAACAGAGAAGTTGAAATGtaa
NLS	CCCAAGAAAAAGCGCAAGGTA **or** CCAAAAAAGAAAAGAAAAGTT **or** CCCAAGAAAAAGAGAAAGGTG (amino acid sequence: PKKKRKV)
HAtag	TACCCATACGACGTCCCAGACTACGCT **or** TATCCTTATGATGTTCCTGATTATGCA
HIStag	CATCATCATCATCATCAC
GS linker	GGAAGT **or** GGATCC **or** GGCTCC (amino acid sequence: GS)
CYC1t_atc	CATGTAATTAGTTATGTCACGCTTACATTCACGCCCTCCCCCCACATCCGCTCTAACCG AAAAGGAAGGAGTTAGACAACCTGAAGTCTAGGTCCCTATTTATTTTTTTATAGTTAT GTTAGTATTAAGAACGTTATTTATATTTCAAATTTTTCTTTTTTTTCTGTACAGACGCG TGTACGCATGTAACATTATACTGAAAACCTTGCTTGAGAAGGTTTTGGGACGCTCGA AGGCTTTAATTTGCAAGCTatc
Tsynth24	TGGGTGGTATGTTATATAACTGTCTAGAAATAAAGAGTATCATCTTTCAAA
ADH1t	GCGAATTTCTTATGATTTATGATTTTTATTATTAAATAAGTTATAAAAAAAATAAGT GTATACAAATTTTAAAGTGACTCTTAGGTTTTAAAACGAAAATTCTTATTCTTGAGT AACTCTTTCCTGTAGGTCAGGTTGCTTTCTCAGGTATAGCATGAGGTCGCTCTTAT TGACCACACCTCTACCGGCATG
SUP4t	TTTTTTTGTTTTTTATGTCT
donor DNA 1 (Combines with sgRNA1, sgRNA2, and sgRNA3)	ttccttggccaactttggttacgactttgtctcacggtgttcaatgtttcGGGCTGATCATTAACTATC CACTGGATGACCAGGATGCCATTGCTGTGGAAGCTGCCTGCACTAATGTTCCAGC ACTCTTTCTTGATGTCTCTGACCAGACACCCATCAACAGTATTATTTTCTCCCATGA AGATGGTACAAGACTGGGTGTGGAGCATCTGGTTGCATTGGGACACaaggaagatg gtaacattttgggtcacaagttagaatacaatttcaactc
donor DNA 2 (Combines with sgRNA4, sgRNA5, and sgRNA6)	atatttacattatggctgttaagcaaaagaacggtattaaggtcaactttGGGCTGATCATTAACTAT CCACTGGATGACCAGGATGCCATTGCTGTGGAAGCTGCCTGCACTAATGTTCCAG CACTCTTTCTTGATGTCTCTGACCAGACACCCATCAACAGTATTATTTTCTCCCATG AAGATGGTACAAGACTGagagatcacatggtcttgttagaatttcgtactgctgctggtatcacttt

Table S3. sgRNA sequences used in this work.

NAME	LOCATION	SPACER SEQUENCE	DIRECT REPEAT	FIGURE
sgRNA1_SpyCas9	250…269	TCATGTTGCTTCATGTGATC	GTTTTAGAGCTAGAAATAGCAAGTTAAAATAAGGCTAGTCCGTTATCAACTTGAAAAAGTGGCACCGAGTCGGTGGTGC	1C, 2C
sgRNA2_SpyCas9	284…303	AACGTAACCTTCTGGCATGG		
sgRNA3_SpyCas9	381…400	AGTCAATAGAATCGAATTGA		
sgRNA4_SpyCas9	523…542	ATCAGACACAACGTTGAAGA		
sgRNA5_SpyCas9	601…620	TGAGAATCTGGTAATAAGAC		
sgRNA6_SpyCas9	636…655	GGTCCTTAGACAAGACAGAT		
non-target sgRNA_SpyCas9	–	AAGTCTTCGTCTGAAGACAA		
sgRNA1_Nme1Cas9	240…263	CCGTTACCCAGATCACATGAAGCA	GTTGTAGCTCCCTTTCTCATTTCGGAAACGAAATGAGAACCGTTGCTACAATAAGGCCGTCTGAAAAGATGTGCCGCAACGCTCTGCCCCTTAAAGCTTCTGCTTTAAGGGGCATCGTTTA	
sgRNA2_Nme1Cas9	286…309	TTCTTGAACGTAACCTTCTGGCAT		
sgRNA3_Nme1Cas9	381…404	AGTCAATAGAATCGAATTGAAGGG		
sgRNA4_Nme1Cas9	530…553	GGACAGAACCGTCTTCAACGTTGT		
sgRNA5_Nme1Cas9	585…608	AATTGGTGACGGTCCAGTCTTATT		
sgRNA6_Nme1Cas9	643…666	CTTTTCATTTGGGTCCTTAGACAA		
non-target sgRNA_Nme1Cas9	–	ACCAGGCCAGCCATGTTTCTGCCA		
sgRNAr1_Nme1Cas9	104…127	AATTCGACCAAAATAGGGACAACA	GTTGTAGCTCCCTTTCTCATTTCGGAAACGAAATGAGAACCGTTGCTACAATAAGGCCGTCTGAAAAGATGTGCCGCAACGCTCTGCCCCTTAAAGCTTCTGCTTTAAGGGGCATCGTTTA	3C
sgRNAr2_Nme1Cas9	134…157	GAGAACTTGTGACCGTTGACATCA		
sgRNAr3_Nme1Cas9	194…217	CAGATGAACTTCAAGGTCAACTTA		
sgRNAr4_Nme1Cas9	223…246	GCCAAGGAACAGGCAACTTACCAG		
sgRNAr5_Nme1Cas9	315…338	CATGGCGGATTTAAAGAAATCATG		
sgRNAr6_Nme1Cas9	347…370	AAGATAGTTCTTTCTTGAACGTAA		

sgRNAr7_Nme1Cas9	406...429	AAGTATCACCTTCGAACTTGACTT		
sgRNAr8_Nme1Cas9	455...478	TTACCATCTTCCTTGAAATCAACA		
sgRNAr9_Nme1Cas9	488...511	TTGAAATTGTATTCTAACTTGTGA		
sgRNAr10_Nme1Cas9	554...577	CTGATTTTAAAGTTGACCTTAATA		
sgRNAr11_Nme1Cas9	596...619	TAGTGGTCAGCTAATTGGACAGAA		
sgRNAr12_Nme1Cas9	624...647	ACCGTCACCAATTGGAGTGTTTTG		
sgRNAr13_Nme1Cas9	730...753	CAGCAGTACGAAATTCTAACAAGA		
sgRNAr14_Nme1Cas9	812...835	GATGATACTCTTTATTTCTAGACA		
sgRNAr1_LwaCas13a	21...48	TGTTTGTGTGTCTATAGAAGTATAGTAA	GATTTAGACTACCCCAAAAACGAAGGGGACTAAAAC	4B, S2, S3B
sgRNAr2_LwaCas13a	76...103	ACACCAGTGAATAATTCTTCACCTTTAG		
sgRNAr3_LwaCas13a	331...358	CTTTCTTGAACATAACCTTCTGGCATGG		
sgRNAr4_LwaCas13a	591...618	GATAATGGTCAGCTAATTGAACAGAACC		
sgRNAr1_RfxCas13d	21...42	TGTGTCTATAGAAGTATAGTAA	CACTAGTGCGAATTTGCACTAGTCTAAAAC (named DR_UrCas13d)	
sgRNAr2_RfxCas13d	76...97	GTGAATAATTCTTCACCTTTAG		
sgRNAr3_RfxCas13d	331...352	TGAACATAACCTTCTGGCATGG		
sgRNAr4_RfxCas13d	591...612	GGTCAGCTAATTGAACAGAACC		
pre-crRNA_LwaCas13a<br>(The underlined sequences in italics are the spacers; the other sequences are the direct repeats)	79...106<br>116...143<br>265...292<br>79...106<br>116...143<br>265...292	GATTTAGACTACCCCAAAAACGAAGGGGACTAAAAC*ACAACACCAGTGAATAATTCTTCACCTT*GATTTAGACTACCCCAAAAACGAAGGGGACTAAAAC*ACCATTAACATCACCATCTAATTCAACC*GATTTAGACTACCCCAAAAACGAAGGGGACTAAAAC*TATCTCGCAAAACATTGAACACCATAAC*GATTTAGACTACCCCAAAAACGAAGGGGACTAAAAC*ACAACACCAGTGAATAATTCTTCACCTT*GATTTAGACTACCCCAAAAACGAAGGGGACTAAAAC*ACCATTAACATCACCATCTAATTCAACC*GATTTAGACTACCCCAAAAACGAAGGGGACTAAAAC*TATCTCGCAAAACATTGAACACCATAAC*		

sgRNAr1_RfxCas13d	21…48	TGTTTGTGTGTCTATAGAAGTA TAGTAA	RfxCas13d(standard DR):<br>AACCCCTACCA ACTGGTCGGGG TTTGAAAC<br><br>RfxCas13d(optimal DR):<br>TACCCCTACCAA CTGGTCGGGGT TTGAAAC	S3A
sgRNAr2_RfxCas13d	76…103	ACACCAGTGAATAATTCTTCAC CTTTAG		
sgRNAr3_RfxCas13d	331…358	CTTTCTTGAACATAACCTTCTG GCATGG		S3C

*The location of each sgRNA represents the target sequence (on either *yEBFP2* or *yEGFP* gene) with respect to the transcription start site of corresponding promoter.

Table S4. Yeast strains engineered in this work.

NAME	GENOTYPE
bYMM584	CEN.PK2-1C (MATa; his3D1; leu2-3_112; ura3-52; trp1-289; MAL2-8c; SUC2)
bYMM1370	bYMM584 pMM1309::TRP1
bYMM1379	bYMM584 pMM1309::TRP1 pMM1303::URA3
bYMM1380	bYMM584 pMM1309::TRP1 pMM1321::URA3
bYMM1381	bYMM584 pMM1309::TRP1 pMM1322::URA3
bYMM1386	bYMM584 pMM1309::TRP1 pMM1303::URA3 pMM1328::LEU2
bYMM1387	bYMM584 pMM1309::TRP1 pMM1303::URA3 pMM1329::LEU2
bYMM1388	bYMM584 pMM1309::TRP1 pMM1303::URA3 pMM1330::LEU2
bYMM1389	bYMM584 pMM1309::TRP1 pMM1322::URA3 pMM1325::LEU2
bYMM1390	bYMM584 pMM1309::TRP1 pMM1322::URA3 pMM1326::LEU2
bYMM1391	bYMM584 pMM1309::TRP1 pMM1322::URA3 pMM1327::LEU2
bYMM1409	bYMM584 pMM1309::TRP1 pMM1303::URA3 pMM1352::LEU2
bYMM1410	bYMM584 pMM1309::TRP1 pMM1303::URA3 pMM1353::LEU2
bYMM1411	bYMM584 pMM1309::TRP1 pMM1303::URA3 pMM1354::LEU2
bYMM1412	bYMM584 pMM1309::TRP1 pMM1322::URA3 pMM1349::LEU2
bYMM1413	bYMM584 pMM1309::TRP1 pMM1322::URA3 pMM1350::LEU2
bYMM1414	bYMM584 pMM1309::TRP1 pMM1322::URA3 pMM1351::LEU2
bYMM1415	bYMM584 pMM1309::TRP1 pMM1321::URA3 pMM1349::LEU2
bYMM1416	bYMM584 pMM1309::TRP1 pMM1321::URA3 pMM1350::LEU2
bYMM1417	bYMM584 pMM1309::TRP1 pMM1321::URA3 pMM1351::LEU2
bYMM1436	bYMM584 pMM1309::TRP1 pMM1303::URA3 pMM1402::LEU2
bYMM1437	bYMM584 pMM1309::TRP1 pMM1322::URA3 pMM1381::LEU2
bYMM1418	bYMM584 pMM1379::TRP1
bYMM1424	bYMM584 pMM1379::TRP1 pMM1321::URA3
bYMM1456	bYMM584 pMM1379::TRP1 pMM1424::URA3
bYMM1458	bYMM584 pMM1379::TRP1 pMM1424::URA3 pMM1426::LEU2
bYMM1459	bYMM584 pMM1379::TRP1 pMM1424::URA3 pMM1427::LEU2
bYMM1460	bYMM584 pMM1379::TRP1 pMM1424::URA3 pMM1428::LEU2
bYMM1461	bYMM584 pMM1379::TRP1 pMM1424::URA3 pMM1429::LEU2
bYMM1462	bYMM584 pMM1379::TRP1 pMM1424::URA3 pMM1430::LEU2
bYMM1463	bYMM584 pMM1379::TRP1 pMM1424::URA3 pMM1431::LEU2

bYMM1464	bYMM584 pMM1379::TRP1 pMM1424::URA3 pMM1445::LEU2
bYMM1465	bYMM584 pMM1379::TRP1 pMM1424::URA3 pMM1453::LEU2
bYMM1466	bYMM584 pMM1379::TRP1 pMM1424::URA3 pMM1446::LEU2
bYMM1467	bYMM584 pMM1379::TRP1 pMM1424::URA3 pMM1447::LEU2
bYMM1468	bYMM584 pMM1379::TRP1 pMM1424::URA3 pMM1448::LEU2
bYMM1469	bYMM584 pMM1379::TRP1 pMM1424::URA3 pMM1454::LEU2
bYMM1470	bYMM584 pMM1379::TRP1 pMM1424::URA3 pMM1455::LEU2
bYMM1471	bYMM584 pMM1379::TRP1 pMM1424::URA3 pMM1380::LEU2
bYMM600	bYMM584 pMM728::LEU2
bYMM666	bYMM584 pMM728::LEU2 pMM813::URA3
bYMM737	bYMM584 pMM728::LEU2 pMM843::URA3
bYMM844	bYMM584 pMM728::LEU2 pMM813::URA3 pMM805::TRP1
bYMM845	bYMM584 pMM728::LEU2 pMM813::URA3 pMM806::TRP1
bYMM846	bYMM584 pMM728::LEU2 pMM813::URA3 pMM792::TRP1
bYMM847	bYMM584 pMM728::LEU2 pMM813::URA3 pMM793::TRP1
bYMM848	bYMM584 pMM728::LEU2 pMM843::URA3 pMM839::TRP1
bYMM849	bYMM584 pMM728::LEU2 pMM843::URA3 pMM840::TRP1
bYMM850	bYMM584 pMM728::LEU2 pMM843::URA3 pMM841::TRP1
bYMM851	bYMM584 pMM728::LEU2 pMM843::URA3 pMM842::TRP1
bYMM852	bYMM584 pMM728::LEU2 pMM813::URA3 pMM888::TRP1
bYMM853	bYMM584 pMM728::LEU2 pMM813::URA3 pMM889::TRP1
bYMM854	bYMM584 pMM728::LEU2 pMM843::URA3 pMM890::TRP1
bYMM855	bYMM584 pMM728::LEU2 pMM843::URA3 pMM891::TRP1
bYMM856	bYMM584 pMM728::LEU2 pMM813::URA3 pMM933::TRP1
bYMM857	bYMM584 pMM728::LEU2 pMM813::URA3 pMM934::TRP1
bYMM858	bYMM584 pMM728::LEU2 pMM843::URA3 pMM935::TRP1
bYMM859	bYMM584 pMM728::LEU2 pMM843::URA3 pMM936::TRP1
bYMM860	bYMM584 pMM728::LEU2 pMM813::URA3 pMM673::TRP1
bYMM957	bYMM584 pMM728::LEU2 pMM843::URA3 pMM995::TRP1
bYMM959	bYMM584 pMM728::LEU2 pMM843::URA3 pMM997::TRP1
bYMM1005	bYMM584 pMM728::LEU2 pMM843::URA3 pMM1062::TRP1
bYMM1006	bYMM584 pMM728::LEU2 pMM843::URA3 pMM1063::TRP1
bYMM1007	bYMM584 pMM728::LEU2 pMM843::URA3 pMM1064::TRP1
bYMM1008	bYMM584 pMM728::LEU2 pMM843::URA3 pMM1065::TRP1

bYMM1010	bYMM584 pMM728::LEU2 pMM843::URA3 pMM995::TRP1 pMM1022::HIS3
bYMM1011	bYMM584 pMM728::LEU2 pMM843::URA3 pMM995::TRP1 pMM1023::HIS3
bYMM1013	bYMM584 pMM728::LEU2 pMM843::URA3 pMM997::TRP1 pMM1022::HIS3
bYMM1014	bYMM584 pMM728::LEU2 pMM843::URA3 pMM997::TRP1 pMM1023::HIS3
bYMM993	bYMM584 pMM728::LEU2 pMM843::URA3 pMM839::TRP1 pMM1022::HIS3
bYMM994	bYMM584 pMM728::LEU2 pMM843::URA3 pMM840::TRP1 pMM1022::HIS3
bYMM995	bYMM584 pMM728::LEU2 pMM843::URA3 pMM890::TRP1 pMM1022::HIS3
bYMM996	bYMM584 pMM728::LEU2 pMM843::URA3 pMM935::TRP1 pMM1022::HIS3
bYMM997	bYMM584 pMM728::LEU2 pMM843::URA3 pMM839::TRP1 pMM1023::HIS3
bYMM998	bYMM584 pMM728::LEU2 pMM843::URA3 pMM840::TRP1 pMM1023::HIS3
bYMM999	bYMM584 pMM728::LEU2 pMM843::URA3 pMM890::TRP1 pMM1023::HIS3
bYMM1000	bYMM584 pMM728::LEU2 pMM843::URA3 pMM935::TRP1 pMM1023::HIS3
